# Molecular detections of 14 amphidomatacean species (Dinophyceae) and their temperature-associated distribution in subarctic waters off eastern Hokkaido, Japan

**DOI:** 10.1093/plankt/fbag029

**Published:** 2026-05-13

**Authors:** Kazuma Nakano, Koyo Kuwata, Takehiro Shimonaka, Urban Tillmann, Taketoshi Kodama, Kazutaka Takahashi, Mitsunori Iwataki

**Affiliations:** Graduate School of Agricultural and Life Sciences, The University of Tokyo, 1-1-1 Yayoi, Bunkyo, Tokyo 113–8657, Japan; Graduate School of Agricultural and Life Sciences, The University of Tokyo, 1-1-1 Yayoi, Bunkyo, Tokyo 113–8657, Japan; Graduate School of Agricultural and Life Sciences, The University of Tokyo, 1-1-1 Yayoi, Bunkyo, Tokyo 113–8657, Japan; Alfred Wegener Institute for Polar and Marine Research, Am Handelshafen 12, Bremerhaven D-27570, Germany; Graduate School of Agricultural and Life Sciences, The University of Tokyo, 1-1-1 Yayoi, Bunkyo, Tokyo 113–8657, Japan; Graduate School of Agricultural and Life Sciences, The University of Tokyo, 1-1-1 Yayoi, Bunkyo, Tokyo 113–8657, Japan; Graduate School of Agricultural and Life Sciences, The University of Tokyo, 1-1-1 Yayoi, Bunkyo, Tokyo 113–8657, Japan

**Keywords:** shellfish poisoning, *Azadinium*, *Amphidoma*, quantitative PCR, eDNA metabarcoding

## Abstract

To better understand the diversity and ecological characteristics of the amphidomatacean dinoflagellates around Japan, their genetic diversity was examined using ITS2-based environmental DNA (eDNA) metabarcoding and cell densities of toxigenic species *Azadinium poporum* and *Az*. *spinosum* were estimated using LSU rDNA-based quantitative PCR (qPCR). Samples were collected in the offshore waters off the coast of Hokkaido, Japan, during the summer in 2023 and 2024. eDNA metabarcoding revealed the presence of 14 species (11 *Azadinium* and three *Amphidoma*), including *Azadinium concinnum* previously not found in the western North Pacific. The presence of *Am. languida* was also detected based on the comparison of ITS phylogeny with the toxigenic strains. Five species predominantly detected in the 2024 cruise exhibited their temperature-associated distribution in the species and intraspecific group (ribotype) levels; *Am. fulgens*, *Az. dexteroporum*, and *Az. spinosum* (ribotype A) were found in the warm-water (warming Kuroshio-Oyashio mixed water), while *Az. dalianense*, *Az. polongum*, and *Az. spinosum* (ribotype C) in the cold-water (surface-warmed subarctic water). Moreover, species-specific qPCR analysis of two toxigenic species revealed the predominant distribution of *Az. spinosum* in the pelagic western North Pacific for the first time, while *Az. poporum* was rarely detected.

## INTRODUCTION

Some species of the marine thecate dinoflagellates *Amphidoma* and *Azadinium*, belonging to the family Amphidomataceae, are known to produce the lipophilic toxin azaspiracids (AZAs), causing diarrhetic shellfish poisoning (Tillmann *et al.,* 2009, 2011, 2012a). Since the discovery of the first AZA-producing species, *Az. spinosum* Elbrächter et Tillmann, its related amphidomataceans have been explored mainly in the Atlantic, and 19 *Azadinium* and 15 *Amphidoma* species have been described to date (Tillmann *et al.,* 2009, 2010, 2011, 2012a, 2012b, 2014, 2018a, 2020, 2025, 2026; Nézan *et al.,* 2012; Luo *et al.,* 2013, 2017; Percopo *et al.,* 2013; Tillmann and Akselman, 2016; Tillmann, 2018; Salas *et al.,* 2021; Kuwata *et al.,* 2023, 2024b, 2025). Among these species, AZAs have been detected in *Az. dexteroporum* Percopo et Zingone, *Az. poporum* Tillmann et Elbrächter, *Az. spinosum*, and *Am. languida* Tillmann, Salas et Elbrächter (Tillmann *et al.,* 2009, 2012a; Krock *et al.,* 2012; Percopo *et al.,* 2013). Production of AZA analogs differs among the intraspecific groups (ribotypes) of these species, for example, in *Az. spinosum*, productions of AZA-1 and -2 in ribotype A, AZA-11 and -51 in ribotype B, and no AZAs in ribotype C, have been reported (Tillmann *et al.,* 2019). Similar ribotype-specific AZA productions are known for *Az. poporum*, e.g. AZA-59 in ribotype A1, no AZAs or AZA-2 in ribotype A2, a variety of AZAs in ribotype B, AZA-2 in ribotype C1, and AZA-2 and -40 in ribotype C2 (Luo *et al.,* 2016; Kuwata *et al.,* 2025). Therefore, it is crucial to identify the toxic amphidomataceans at the ribotype level to comprehend their ecological characteristics and mitigate shellfish poisonings effectively.

In the Pacific, recent explorations have shown a high species diversity of the Amphidomataceae, and *Az. anteroporum* Kuwata, Takahashi, Lum et Iwataki, *Az. dalianense* Luo, Gu et Tillmann, *Az. fusiforme* Kuwata, Tillmann, Kim, Takahashi et Iwataki, *Az. inconspicuum* Kuwata, Lum, Takahashi et Iwataki, *Az. zhuanum* Luo, Tillmann et Gu, and *Am. fulgens* Kuwata, Takahashi, Lum, Benico et Iwataki were newly described (Luo *et al.,* 2013, 2017; Kuwata *et al.,* 2023, 2024b, 2025; Tillmann *et al.,* 2026). The four AZA-producing species have also been found in the Pacific (Potvin *et al.,* 2012; Gu *et al.,* 2013; Takahashi *et al.,* 2021; Kuwata *et al.,* 2024a, 2025), and AZAs were detected from the Pacific strains of *Az. poporum*, *Az. spinosum*, and *Am. languida* (Krock *et al.,* 2012; Ozawa *et al.,* 2021, 2023, 2024; Takahashi *et al.,* 2021; Kuwata *et al.,* 2024a, 2025). *Azadinium poporum* has been found widely across the coastal waters of the Pacific, in Chile, China, Japan, Korea, New Zealand, the United States, and Vietnam, with detections of a variety of AZA analogs (Potvin *et al.,* 2012; Gu *et al.,* 2013; Krock *et al.,* 2014, 2019; Smith *et al.,* 2016; Kim *et al.,* 2017; Tillmann *et al.,* 2017; Ozawa *et al.,* 2021, 2023, 2024; Takahashi *et al.,* 2021; Liu *et al.,* 2023; Kuwata *et al.,* 2025). On the other hand, the other three toxigenic amphidomataceans were rare in the Pacific. For *Az. spinosum*, only one toxigenic strain was isolated from the Seto Inland Sea, Japan (Kuwata *et al.,* 2025; Ozawa *et al.,* 2025). For *Az. dexteroporum*, only one non-toxigenic strain was found in the western North Pacific (Kuwata *et al.,* 2025). For *Am. languida*, one toxigenic strain from the Mexican Pacific and another non-toxigenic strain from New Zealand have been reported (Balci *et al.,* 2023; Kuwata *et al.,* 2024a).

The morphology and AZA production of amphidomatacean species are becoming unveiled, but their distribution and ecological traits remain largely unexplored. For example, germinated cells were obtained in incubated seabed sediments only from *Az. cuneatum* Tillmann et Nézan, *Az. dalianense*, *Az. obesum* Tillmann et Elbrächter, *Az. poporum*, and *Az. zhuanum* (Potvin *et al.,* 2012; Luo *et al.,* 2013, 2016, 2017; Tillmann *et al.,* 2016; Kim *et al.,* 2017; Liu *et al.,* 2023), but resting cysts or cyst-like cells have been observed only in *Az. poporum*, *Az. polongum* Tillmann, and *Az*. *zhuanum* (Tillmann *et al.,* 2012b; Gu *et al.,* 2013; Luo *et al.,* 2017), and is yet unknown in other amphidomataceans. Although morphological identification of the tiny motile cells is challenging, recent application of molecular detections from environmental DNA (eDNA) has enabled us to assess the diversity and cell density of amphidomataceans in seawater. High species diversity has been revealed using eDNA metabarcoding in the China Sea and adjacent seas (e.g. Fu *et al.,* 2021; Wang *et al.,* 2022; Liu *et al.,* 2023; Tao *et al.,* 2025; Cui *et al.,* 2026). In the Taiwan Strait, 11 *Azadinium* species were detected by ITS1 and LSU rDNA-based eDNA metabarcoding, including three species previously unreported in the western North Pacific, *Az*. *obesum*, *Az*. *perfusorium* Tillmann et Salas, and *Az*. *polongum* (Liu *et al.,* 2023). In the China Sea, long-read metabarcoding of ITS and LSU rDNA detected 17 species, including *Am*. *parvula* Tillmann et Gottschling and *Az*. *perforatum* Tillmann, Wietkamp et Gu, which had previously been undetected in the western North Pacific (Cui *et al.,* 2026). In surface sediments of the East China Sea, *Az*. *galwayense* Salas et Tillmann and *Az. spinosum* were recently detected by LSU rDNA-based eDNA metabarcoding, as well as five other *Azadinium* that the cysts or germinated cells were previously reported (Tao *et al.,* 2025). ITS- and LSU rDNA-based eDNA metabarcoding detected the ribotype B of *Az*. *poporum* and ribotype A of *Az*. *spinosum* in the Gulf of Thailand, *Az*. *poporum* (ribotypes A–C) and *Az*. *spinosum* (ribotypes A and B) in the Taiwan Strait, and multiple ribotypes of *Az*. *dalianense* (Fu *et al.,* 2021; Liu *et al.,* 2023; Chai *et al.,* 2026; Cui *et al.,* 2026). In the Japanese coastal area and adjacent seas, *Az*. *dexteroporum*, *Az*. *poporum*, *Az*. *spinosum*, and *Az*. *trinitatum* Tillmann et Nézan were detected by SSU rDNA-based eDNA metabarcoding (Sildever *et al.,* 2019, 2022; Wu *et al.,* 2024; Funaki *et al.,* 2025). Cell densities of toxigenic amphidomataceans have been estimated by eDNA species-specific quantitative PCR (qPCR), especially for toxigenic species (Toebe *et al.,* 2013; Wietkamp *et al.,* 2019a, 2019b). In the North Sea and adjacent seas, high cell densities of *Az*. *spinosum* reaching peak densities of 83 000 cells L^−1^ were detected by qPCR (Wietkamp *et al.,* 2020). In the Pacific coast of the United States, *Az*. *poporum* and *Az*. *spinosum* were detected with peak densities of 10 525 and 156 cells L^−1^, respectively (Adams *et al.,* 2020). In the Taiwan Strait, the qPCR assay showed low abundance of *Az*. *poporum* up to 209 cells L^−1^ (Liu *et al.,* 2023).

In the western North Pacific region, the diversity of amphidomatacean dinoflagellates has been explored at species and ribotype levels, primarily in the coastal waters of tropical and subtropical regions (e.g. Fu *et al.,* 2021; Liu *et al.,* 2023). To delve deeper into the diversity and distribution of amphidomataceans in the subarctic offshore waters, this study conducted eDNA sampling off the coast of eastern Hokkaido, Japan, a region where the cold and warm water currents meet. During the eDNA detection efforts in August 2023, unexpected high cell densities of *Az. spinosum* were observed. Subsequently, in September 2024, ITS2-based eDNA metabarcoding was conducted in the region. Cell densities of two toxigenic species, *Az. poporum* and *Az*. *spinosum* were also estimated by species-specific LSU rDNA-based qPCR analysis.

## METHODS

### Sampling and sample processing

**Fig. 1:**
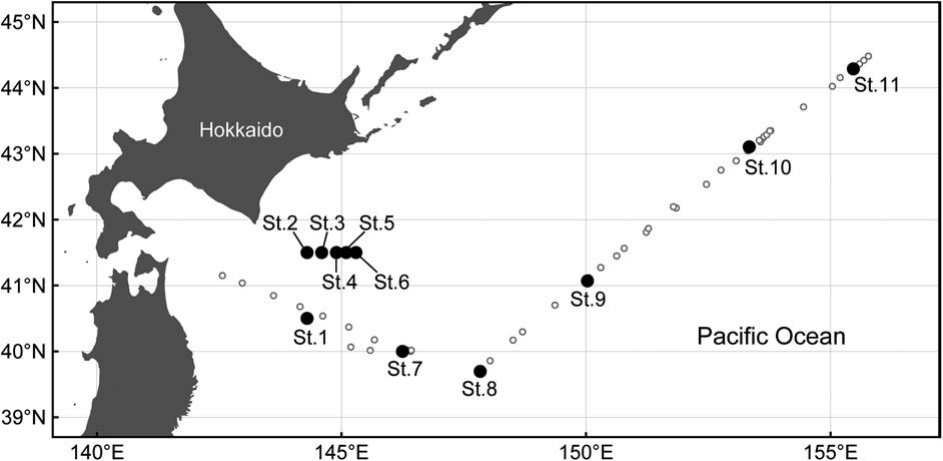
Sampling locations of seawater for environmental DNA extraction, off eastern Hokkaido, Japan, in August 2023 (St. 1–6) and in September 2024 (St. 7–11). Small white circles show locations where surface seawaters were collected using the automated seawater filtration system in September 2024.

Seawater samples for eDNA extractions were collected from off eastern Hokkaido in August 2023 and September 2024 (Fig. 1, Table S1) by the R/V Shinsei-maru research cruises. Following a preliminary survey in 2023, a larger number of samples covering wider environmental conditions were collected in 2024. In 2023, surface seawater was collected using a bucket at St. 1–6, and additional samples were collected at St. 2 and St. 4, from 20 m depth and subsurface chlorophyll maximum (SCM) layer using Niskin-X sampling bottles. In 2024, 65 vertical seawater samples were collected at St. 7–11 from 12 depths (0, 10, 20, 30, 40, 50, 60, 80, 100, 125, 150, and 200 m) and an additional layer around SCM. Water temperature and salinity were measured by a CTD (Sea-Bird Electronics, Washington, USA) or a portable electrical conductivity meter D-220C (Horiba, Kyoto, Japan). For eDNA extraction, 1 L of seawater samples was filtered through a 3.0 μm pore-size Isopore membrane filter (Millipore, Eschborn, Germany) for efficient collection of small dinoflagellate cells after the pre-filtration using a 100 μm mesh. Samples were stored at −40°C and transported to the laboratory. Environmental DNAs were extracted from the particles on the filters by using a DNeasy Plant Mini Kit (Qiagen, Hilden, Germany). For measuring the concentration of chlorophyll *a*, particles in seawater were collected on a glass fiber filter (GF/F, Whatman, Maidstone, UK) and measured by fluorometers using the methods of Welschmeyer (1994).

In 2024, a total of 50 surface seawater samples were also collected from the ship’s bottom using an automated seawater filtration system (Toyo Roshi Kaisha; Japanese Patent No. 6484572) (Fig. S1, Table S2), and filtered through a 0.2 μm pore-size membrane for the eDNA availability to other studies such as detection of prokaryotes. Water temperature and salinity were measured by Automated Marine Environment Monitor for Biogeochemical Observations (Tsuda *et al.,* 1993). Samples were stored at −40°C and transported to the laboratory. Environmental DNAs were extracted from the particles on the filters by using the method according to Okazaki *et al.* (2023).

### Culture strain

To compare the morphology and phylogenetic positions of amphidomatacean cells inhabiting off eastern Hokkaido, we attempted to establish culture strains by micropipetting, but only one strain was cultivated. A strain of *Az*. *spinosum* (NK0178) was isolated from 0 m depth of St. 8 (39.7°N, 147.8°E), under an inverted microscope CKX53 (Olympus, Tokyo, Japan). The culture was maintained in half-strength IMK medium (Wako, Tokyo, Japan), with a salinity of 30 at 23°C under a 12:12 h light: dark cycle regime. ITS and LSU rDNA sequences (LC921252) were determined according to Iwataki *et al.* (2022) with slight modifications of annealing temperatures of the first and second PCR to 55°C and 51°C, respectively.

For SEM observation, cells were fixed in 2% OsO_4_ (w/v) solution on a poly-L-lysine-coated SEM plate for 15 min at room temperature, then rinsed twice with distilled water for 15 min each. Cells were rinsed in a dilution series of filtered seawater (80, 60, 40, 20%) for 10 min each, rinsed three times with distilled water for 10 min each, dehydrated in an ethanol series (30, 50, 75, 90, 95, and 99.5%) for 10 min each, and finally replaced with an isoamyl acetate for 15 min. Cells were dried using a JCPD-5 critical point dryer (JEOL, Tokyo, Japan) and sputter-coated with platinum palladium before being observed using an S-4800 SEM (Hitachi, Tokyo, Japan) operated at 5.0 kV. The length and width of cells were measured using SEM images. NK0178 strain was lost before the other morphological observations and toxin analysis.

### eDNA sequencing

DNA sequences of ITS2 region were amplified from extracted eDNA using dinoflagellate-specific primers ITS300F and D1R-reverse (Scholin *et al.,* 1994; Fu *et al.,* 2021). The first PCR was conducted in a cocktail, which contained 10 μL of the KOD One PCR Master Mix (Toyobo, Osaka, Japan), 6.0 pmol of each primer, and 2 μL of template environmental DNA, with the final volume adjusted to 20 μL using sterilized distilled water. The thermal conditions were 98°C for 1 min, followed by 35 cycles of 98°C for 10 s, 47°C for 5 s, 68°C elongation for 10 s, and final extension of 2 min at 68°C. The amplicons were purified using AMpure XP (Beckman Coulter, California, USA). The second PCR was performed using Nextra XT Index kit (Illumina, California, USA), and conducted in a cocktail, which contained 10 μL of KOD One Master mix, 6.0 pmol of index forward and reverse primers, and 2 μL of the purified first PCR amplicon, with the final volume adjusted to 20 μL using sterilized distilled water. The amplicons were purified using AMpure XP, and the samples were mixed at equimolar concentrations. The libraries were sequenced on an Illumina MiSeq using MiSeq Reagent Kit v3 (paired-end reads; 2 × 300 bp).

### Phylogenetic analysis

The obtained sequences by MiSeq were processed using the R software package DADA2 (Callahan *et al.,* 2016; Callahan *et al.,* 2017). The primer sequences were removed using cutadapt 4.9 (Martin, 2011), and the sequences were quality filtered, merged, dereplicated, and chimeras removed through the DADA2 ITS Pipeline Workflow (1.8) to identify amplicon sequence variants (ASVs). The value for truncQ in filterAndTrim function of the workflow was changed to 10. Numbers in ASV names were arranged in descending order of the sequence detections; a smaller number indicates a higher read number across all samples. Taxonomic assignment for the ASVs was performed using BLAST search with a database consisting of 15 706 sequences downloaded with query “Dinophyceae[organism] AND (ITS2[title] OR internal transcribed spacer 2[title])” from GenBank (https://www.ncbi.nlm.nih.gov/) on 25 April 2025. Each ASV was annotated to the dinoflagellate families based on the highest e-value in the respective reference database, with critical levels of 90% identity rate and 80% query cover rate. The ASVs which assigned to the family Amphidomataceae were used for subsequent analysis.

The sequences of ITS region (ITS1–5.8S-ITS2) of the Amphidomataceae were retrieved from GenBank and aligned using the MAFFT v7.110 online program (Katoh *et al.,* 2019) with default settings. The outgroups selected were *Heterocapsa rotundata* (Lohmann) Hansen (PQ645505) and *H*. *steinii* Tillmann, Gottschling, Hoppenrath, Kusber et Elbrächter (MF423346). The alignment was trimmed from primer ITSA to ITSB range (Sato *et al.,* 2011) and aligned with ASVs using the MAFFT with —addfragments settings. The multiple alignment was manually checked with AliView v1.28 (Larsson, 2014). Four ASVs, positioned outside the amphidomatacean clade, were removed.

Phylogenetic trees were constructed based on maximum likelihood (ML) using MEGA 10 (Kumar *et al.,* 2018; Stecher *et al.,* 2020) with the selected best substitution model: General time reversible (GTR) + gamma distribution (G = 0.5360) + proportion of invariable sites (I = 0.1370). All sites, including those with missing data and sequence alignment gaps, were used in the analysis (using the “Use all sites” option). Bootstrap support values were estimated using 1000 replicates. Posterior probabilities of Bayesian inference (BI) were calculated using MrBayes v.3.2.7a (Ronquist *et al.,* 2012). Four Markov chain Monte Carlo chains were run for 5 000 000 generations, and trees were sampled every 100 generations with a burn-in of the first 25% generations. The substitution model used for BI was GTR + G (0.7093) + I (0. 2351), selected based on the lowest AIC scores using PAUP* v.4.0a and MrModeltest2 v.2.4 (Swofford, 2003; Nylander, 2004). Convergence of the chains was confirmed when the average standard deviations of the split frequencies were below 0.01 after calculations. Each ASV was identified to species or ribotype within the Amphidomataceae based on its phylogenetic position in the tree.

### Quantitative PCR

Species-specific primers and TaqMan probes of *Az*. *poporum* and *Az*. *spinosum* targeting LSU rDNA (D1–D3) were newly designed, except for the reverse primer for *Az*. *spinosum* (Table I). To confirm species-specific reactions, primer/probe cross-reactivity was tested for amphidomatacean strains and a strain of *Heterocapsa* sp. (Table S3). qPCR analysis was performed on a QuantStudio 1 (ThermoFisher Scientific, MA, USA). Each reaction contained 2.0 μL of template DNA, 0.6 μL each of forward and reverse primers (10 μM), 0.5 μL of TaqMan probe (10 μM), 10 μL of TaqPath BactoPure Microbial Detection Master Mix (ThermoFisher Scientific), with autoclaved Milli-Q water added to a final volume of 20 μL. The thermal cycle conditions followed those described by Toebe *et al.* (2013). Quantification cycle (Cq) values were averaged from two technical replicates. Total DNAs of *Az. poporum* ribotype C1 (LEtAz168 strain) and *Az. spinosum* ribotype A cultures (HrAz562 strain), adjusted to 20 000 cells, were extracted in the same manner as field samples. Ten-fold serial dilutions were used to generate standard curves at each qPCR run. The limit of detection and quantification were estimated to be approximately two cell equivalents per reaction. Calibration curves were shown in Fig. S4 with amplification efficiencies of ~100–110% and R^2^ values ranging from 0.99 to 1.00. Cell numbers of the two species in seawater samples were estimated from the same eDNA extraction used also for metabarcoding analysis.

**Table I:** Species-specific primers and probes to Azadinium poporum and Az. spinosum used for qPCR in this study

	TaqMan probe (5′–3′)	Forward primer (5′–3′)	Reverse primer (5′–3′)	Reference
*Azadinium poporum*	Apop596T GTGTTTGAGTC GTCTGGAAGC	Apop569F GTATCGATCCC TGCGTGTC	Apop651R GRACTTTCTRGG CWCCTYGAGC	This study
*Azadinium spinosum*	Asp653T AGTCCTTYTGG GCGCCCTGAG	Asp599F TTTGGGTCGCC TGGAAAC	Asp48F TCGTCTTTGTGT CAGGGAGATG	Toebe *et al.* (2013), This study

### Statistical analysis

Statistical analyses were conducted using the R software (R Core Team, 2023). The Kruskal-Wallis test was used to test for significant differences in detected temperature between each ribotype of *Az*. *spinosum* (α = 0.01) and the Dunn’s test with Holm’s *p*-value adjustment was used for follow-up pairwise testing. Linear regression analysis was conducted between ASV read numbers obtained and the cell densities. The data used in this study are available in Figshare (DOI: 10.6084/m9.figshare.31148959).

## RESULTS

### Hydrography

**Fig. 2:**
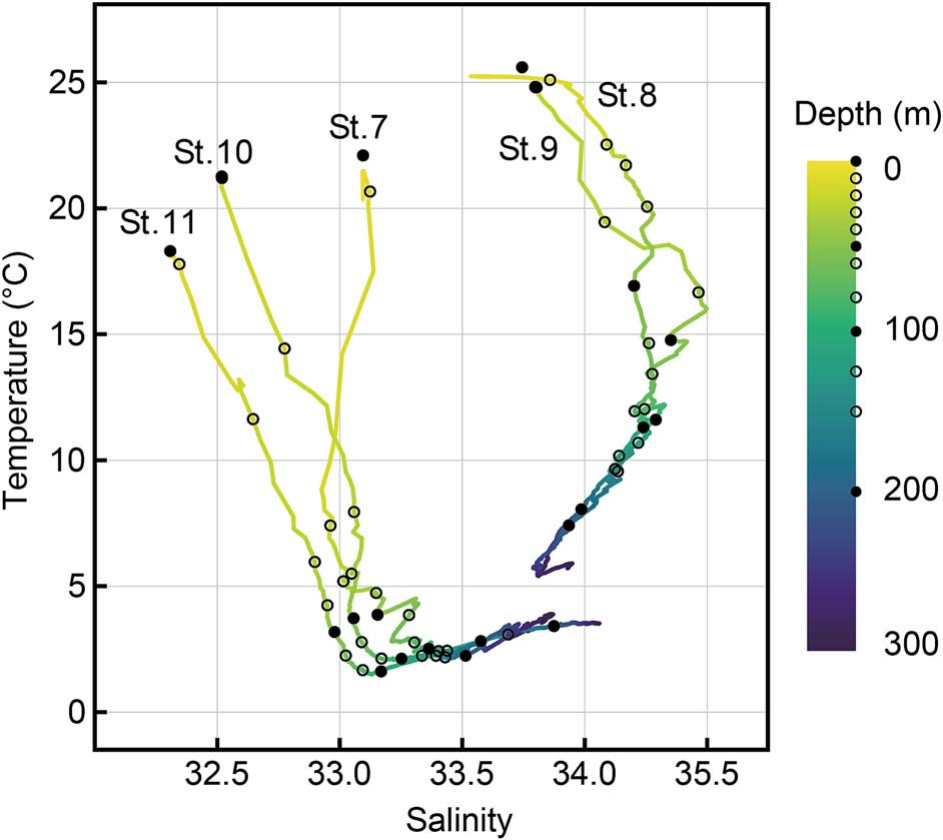
Temperature-salinity diagram of 0–300 m depths in September 2024. Black (0, 50, 100, 200 m) and white circles (other depths) indicate 12 depths of seawater samples for environmental DNA extraction. Note temperature difference of sampling stations, cold-waters (St. 7, 10, 11) and warm-waters (St. 8, 9).

During the 2024 cruise, temperature and salinity upper 300 m were 1.6–25.6°C and 32.3–34.5 for St. 7–11, and 19.0–25.9°C and 32.1–34.4 for surface seawater samples, respectively (Fig. 2). Five stations in 2024 were distinguished into the cold-water mass (St. 7, 10, 11) and warm-water mass (St. 8, 9). Temperatures at 50–200 m depth were below 5°C at cold-water stations and above this threshold at warm-water stations (Fig. 2). During the 2023 research cruise, water temperature and salinity in the upper 300 m at St. 1–6 ranged from 2.4–24.6°C and 32.8–34.4, respectively (Fig. S2). The temperature and salinity profiles of six stations in 2023 were similar to each other and were unable to be distinguished using the above criteria.

### eDNA metabarcoding

**Fig. 3:**
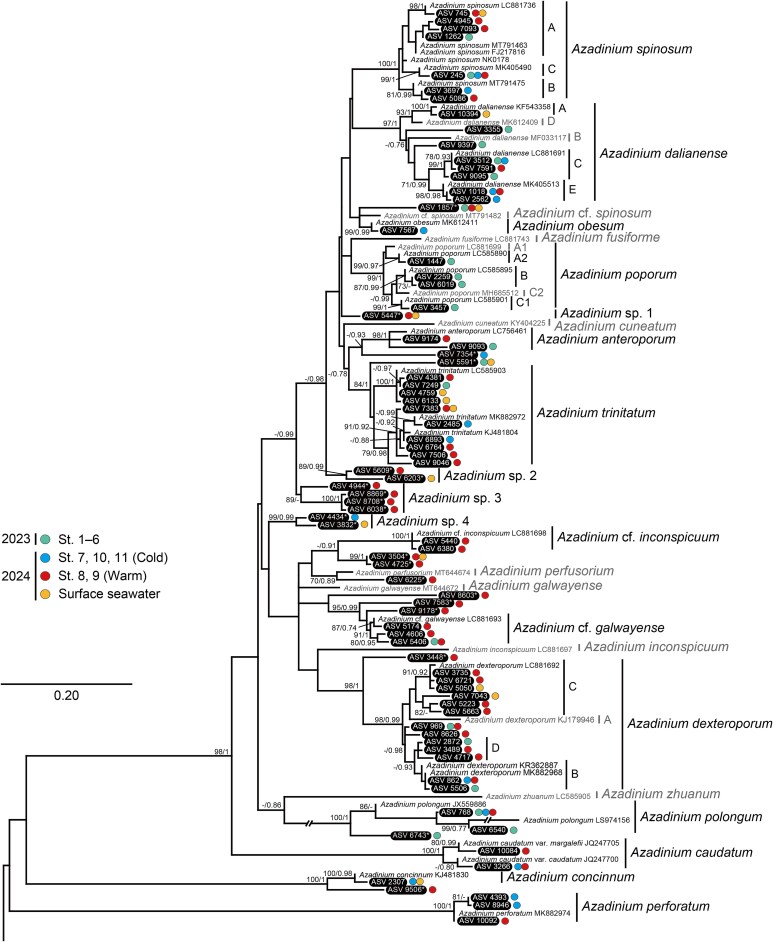
ML phylogeny of *Azadinium* inferred from ITS2 sequences. Bootstrap support values (≥ 70%) of ML and posterior probabilities (≥ 0.70) of Bayesian inference are shown at nodes. ASVs detected in this study are highlighted in black. Asterisks show the unidentified ASVs not assigned to known *Azadinium* clades. Colored circles indicate seawater samples for each ASV.

**Fig. 4:**
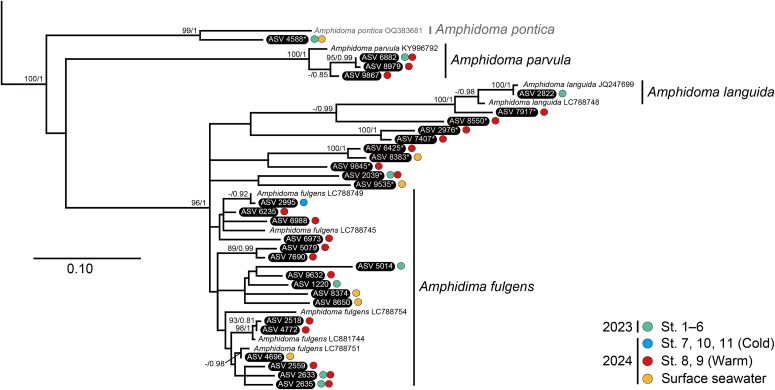
ML phylogeny of *Amphidoma* inferred from ITS2 sequences. Bootstrap support values (≥ 70%) of ML and posterior probabilities (≥ 0.70) of Bayesian inference are shown at nodes. ASVs detected in this study are highlighted in black. Asterisks show the unidentified ASVs not assigned to known *Amphidoma* clades. Colored circles indicate seawater samples for each ASV.

**Fig. 5:**
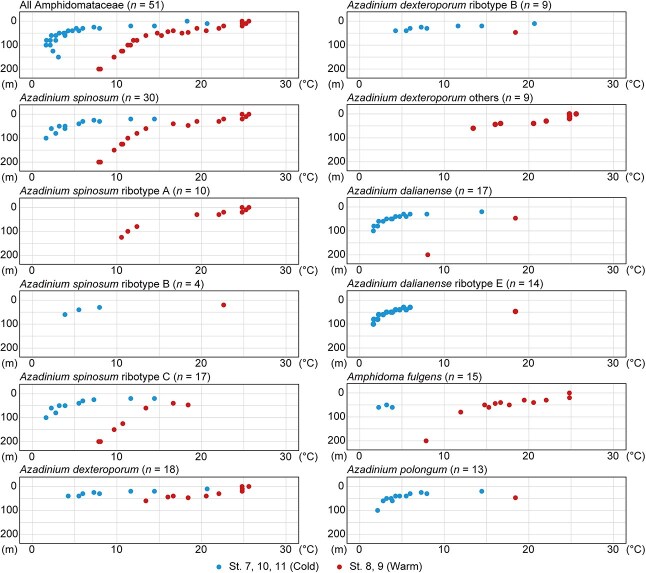
Depth and temperature of five amphidomatacean dinoflagellates predominantly found in vertical seawater samples collected in 2024. Numbers (*n*) associated with species and ribotype names indicate the detected sample numbers. Blue dots are detection in cold-water (St. 7, 10, 11) and red dots are in warm-water (St. 8, 9).

A total of 10 865 ASV sequences (7 875 794 reads) of dinoflagellates were obtained from a total of 125 eDNA samples, of which 1759 ASVs were annotated to the Dinophyceae and 110 ASVs to the Amphidomataceae. The rarefaction curves were roughly saturated for all samples (Fig. S3). The amphidomatacean ASVs were identified based on the positions and related species in the ITS phylogeny (Fig. S5). The originating seawaters for each ASV were differentiated for the water in 2023 (St. 1–6), cold-water (St. 7, 10, 11), warm-water (St. 8, 9), and surface seawater in 2024 (Figs 3–5). In 10 samples of the mixed-water (St. 1–6) in 2023, 9268 reads of 29 ASVs were detected (see the data in Figshare). In 50 surface seawater samples in 2024, 2241 reads of 20 ASVs were detected. In 39 samples in the cold-water (St. 7, 10, 11) in 2024, 6764 reads of 17 ASVs were detected. In 26 samples of the warm-water (St. 8, 9) in 2024, 4081 reads of 67 ASVs were detected.

A total of 79 ASV sequences were assigned to the *Azadinium* clade in the ITS phylogeny (Fig. 3), of which 58 ASVs were nested within the clades of 11 *Azadinium* species, *Az. anteroporum* (2 ASVs), *Az. caudatum* (Halldal) Nézan et Chomérat (2), *Az. concinnum* Tillmann et Nézan (1), *Az. dalianense* (8), *Az. dexteroporum* (13), *Az. obesum* (1), *Az. perforatum* (3), *Az. polongum* (2), *Az. poporum* (4), *Az. spinosum* (7), and *Az. trinitatum* (10). Within the clades of *Az. spinosum*, *Az. dalianense*, *Az. poporum*, and *Az. dexteroporum*, ASVs were assigned to multiple ribotypes. ASVs of *Az. spinosum* were represented by ribotypes A (4 ASVs), B (2), and C (1). In *Az*. *dalianense*, ASVs corresponded to ribotypes A (1), C (3), and E (2), while the positions of the other two ASVs were unclear. In *Az*. *poporum*, ASVs were assigned to ribotypes A2 (1), B (2), and C1 (1), and all were detected in 2023. In *Az*. *dexteroporum*, ASVs corresponded to ribotypes B (2), C (6), and D (3), and the positions of two ASVs were unclear. Ribotype D of *Az*. *dexteroporum* was newly defined in this study. For previously identified clades of undescribed *Azadinium* species, ASVs were positioned in *Az.* cf. *inconspicuum* (2) and *Az.* cf. *galwayense* (3). Other ASVs, indicated with asterisks in Fig. 3, were unidentified ASVs not assigned to known clades. Some of the unidentified ASVs were closest to *Az. obesum*/*Az.* cf. *spinosum* (1), *Az. anteroporum* (1), *Az. trinitatum* (1), *Az.* cf. *inconspicuum* (2), *Az. perfusorium* (1), *Az.* cf. *galwayense* (3), *Az. dexteroporum* (1), *Az. polongum* (1), *Az*. *concinnum* (1). Other unidentified ASVs branched independently were named *Azadinium* sp. 1–4 in this study. *Azadinium* sp. 1 (1) showed a multifurcated topology, with one clade consisting of *Az*. *fusiforme* and *Az*. *poporum*, and another clade comprising *Az. dalianense*, *Az. obesum*, and *Az. spinosum*. *Azadinium* sp. 2 (2) and sp. 3 (4) were paraphyletic to the clade, which consisted of seven *Azadinium* species, including *Az. spinosum* and *Az. trinitatum*. *Azadinium* sp. 4 (2) was deeply branched at the base of the clade including *Az. dexteroporum*, *Az. galwayense*, *Az. inconspicuum*, and *Az. perfusorium*.

Thirty-one ASV sequences were assigned to the *Amphidoma* clade in the ITS phylogeny (Fig. 4). A total of 21 ASVs were positioned in the clades of three *Amphidoma* species, *Am. parvula* (3), *Am. languida* (1), and *Am. fulgens* (17). The other 10 ASVs, indicated with asterisks in Fig. 4, were unidentified *Amphidoma* ASVs. An unidentified ASV 4588 was related to *Am*. *pontica* Tillmann et Dzhembekova, while the sequence differed from the species (OQ383681) 8.3%. The remaining nine ASVs were positioned in a clade consisting of *Am*. *fulgens* and *Am*. *languida*, but they could not be confidently assigned to either species.

In 65 vertical samples collected during the 2024 cruise, amphidomatacean ASVs were found in 51 samples throughout the water depth range of 0–200 m and the temperature range of 1.6–25.6°C (Table II, Fig. 5). Five amphidomatacean species were detected in more than 10 samples. *Azadinium spinosum* (*n* = 30) was detected from a wide temperature range (1.6–25.6°C, mean 13.8°C). In *Az. spinosum*, the ribotype A (*n* = 10) was found in the warm-water (10.5–25.6°C, mean 19.9°C). Two ASVs of the ribotype B (*n* = 3) were detected in the different waters; the ASV 3697 in the cold-water (3.9–7.9°C, mean 5.8°C), and the ASV 5086 in the warm-water (22.6°C). The ribotype C (*n* = 10) was found in the cold-water (1.6–14.4°C, mean 5.8°C) and warm-water (7.9–18.4°C, mean 12.1°C). Water temperatures where the ribotypes A or C detected differed significantly (*P* < 0.01). *Azadinium dexteroporum* (*n* = 18) was detected in a temperature range of 4.2–25.6°C (mean 15.8°C). Most ASVs were found in the warm-water (13.4–25.6°C, mean 20.7°C), while the ribotype B (*n* = 9) was found mainly in the cold-water (4.2–20.7°C, mean 10.7°C). *Azadinium dalianense* (*n* = 14) was found mainly in the cold-water (1.6–18.4°C, mean 5.6°C), of which many ASVs were assigned to the ribotype E (*n* = 17) mainly found in the cold-water (1.6–6.0°C, mean 3.6°C), except one sample in the warm-water (18.4°C). *Amphidoma fulgens* (*n* = 15) was found at temperature of 2.2–24.8°C (mean 14.7°C), mainly in the warm-water (7.9–25.6°C, mean 17.7°C), although ASV 2995 was exceptionally found in the cold-water (2.2–3.9°C, mean 3.1°C). *Azadinium polongum* (*n* = 13) was detected in a temperature range of 2.1–18.4°C (mean 6.5°C), mainly in the cold-water (2.1–14.4°C, mean 5.4°C), while the ASV 768 was also found in the warm-water (18.4°C).

**Table II:** Detected sample numbers (n) of Amphidoma and Azadinium species in 2023 and 2024 cruises. ASV read numbers are given in brackets, with mean temperature and temperature range in brackets. n.d., no detection

Species	2023 (*n* = 10)	2024 vertical (*n* = 65)	2024 surface (*n* = 50)	Reports in western North Pacific
*Am*. *fulgens*	*n* = 5 (1069), 21.3 (15.9–24.6)°C	*n* = 15 (1098), 14.7 (2.2–24.8)°C	*n* = 3 (101), 24.6 (22.6–25.7)°C	China^1^, Japan^2,3^, Malaysia^2^, Philippines^3^
*Am*. *languida*	*n* = 1 (192), 11.8°C	*n* = 1 (18), 16.7°C	n.d.	China^1^, China(?)^4,5^, Japan(?)^6^
*Am*. *parvula*	*n* = 1 (14), 24.6°C	*n* = 3 (22), 24.3 (22.6–25.6)°C	n.d.	China^1^
*Az*. *anteroporum*	*n* = 1 (9), 24.6 °C	*n* = 1 (9), 25.6°C	n.d.	China^1^, Japan^7^
*Az*. *caudatum* var. *caudatum*	n.d.	*n* = 2 (148), 11.6 (5.5–17.7)°C	n.d.	Japan^3^, Thailand^8^
*Az*. *caudatum* var. *margalefii*	n.d.	*n* = 1 (4), 22.1°C	n.d.	China^1,9^, Japan^3^, Thailand^8^
*Az*. *concinnum*	n.d.	*n* = 2 (233), 12.8 (7.3–18.3)°C	*n* = 1 (39), 20.2°C	
*Az*. *dalianense*	*n* = 4 (192), 20.5 (11.8–24.6)°C	*n* = 17 (1375), 5.6 (1.6–18.4)°C	*n* = 1 (3), 23.2°C	China^1,9,10,11,12^, Japan^3^
Ribotype A	n.d.	n.d.	*n* = 1 (3), 23.2 °C	
Ribotype C	*n* = 2 (45), 17.1 (11.8–22.4)°C	*n* = 3 (114), 10.1 (7.9–14.4)°C	n.d.	
Ribotype E	n.d.	*n* = 14 (1261), 4.7 (1.6–18.4)°C	n.d.	
*Az*. *dexteroporum*	*n* = 6 (1220), 21.5 (15.9–24.6)°C	*n* = 18 (1922), 15.8 (4.2–25.6)°C	*n* = 2 (90), 22.7 (22.6–22.9)°C	China^1,4,5,9,13^, Japan^3,14,15^, Thailand^8^
Ribotype B	*n* = 1 (51), 21.1°C	*n* = 9 (1378), 10.7 (4.2–20.7)°C	n.d.	
Ribotype C	n.d.	*n* = 5 (252), 20.9 (16.0–24.8)°C	*n* = 2 (90), 22.7 (22.6–22.9)°C	
Ribotype D	*n* = 1 (186), 15.9°C	*n* = 3 (203), 22.4 (16.7–25.6)°C	n.d.	
*Az*. cf. *galwayense*	*n* = 1 (18), 15.9°C	*n* = 6 (169), 23.2 (19.5–25.6)°C	n.d.	Japan^3^
*Az*. cf. *inconspicuum*	n.d.	*n* = 3 (89), 23.3 (22.1–25.3)°C	n.d.	Japan^3^
*Az*. *obesum*	n.d.	*n* = 1 (21), 2.8°C	n.d.	China^1,9,10,12^
*Az*. *perforatum*	n.d.	*n* = 6 (96), 5.1 (1.7–12.4)°C	n.d.	China^1^
*Az*. *polongum*	*n* = 2 (66), 16.5 (11.8–21.1)°C	*n* = 13 (1569), 6.5 (2.1–18.4)°C	n.d.	China^1,9,10,12,16^
*Az*. *poporum*	*n* = 5 (1057), 20.9 (12.0–24.6)°C	n.d.	n.d.	China^1,8,9,12,17^, Japan^3,6,14,18^, Korea^19^
Ribotype A2	*n* = 2 (598), 22.4°C	n.d.	n.d.	
Ribotype B	*n* = 4 (326), 20.5 (12.0–24.6)°C	n.d.	n.d.	
Ribotype C1	*n* = 1 (133), 22.4°C	n.d.	n.d.	
*Az*. *spinosum*	*n* = 3 (4908), 16.3 (11.8–21.1)°C	*n* = 30 (2636), 12.0 (1.6–25.6)°C	*n* = 7 (1310), 23.9 (22.6–25.7)°C	China^1,8,9,11,12^, Japan^3,6,15^
Ribotype A	*n* = 2 (751), 18.5 (15.9–21.1)°C	*n* = 10 (445), 19.9 (10.5–25.6)°C	*n* = 7 (1310), 23.9 (22.6–25.7)°C	
Ribotype B	n.d.	*n* = 4 (179), 10.0 (3.9–22.6)°C	n.d.	
Ribotype C	*n* = 2 (4157), 16.5 (11.8–21.1)°C	*n* = 17 (2012), 8.4 (1.6–18.4)°C	n.d.	
*Az*. *trinitatum*	*n* = 1 (24), 24.6 °C	*n* = 9 (422), 12.8 (1.6–25.6)°C	*n* = 3 (126), 24.7 (23.2–25.7)°C	China^1,9,20^, Japan^18,21^, Thailand^8^
*Azadinium* sp. 1	n.d.	*n* = 1 (17), 22.6°C	*n* = 1 (36), 22.6°C	
*Azadinium* sp. 2	n.d.	*n* = 1 (49), 9.7°C	*n* = 1 (39), 25.7°C	
*Azadinium* sp. 3	n.d.	*n* = 5 (129), 21.6 (16.0–25.6)°C	n.d.	
*Azadinium* sp. 4	n.d.	*n* = 1 (81), 7.9 °C	*n* = 2 (110), 22.9 (22.6–23.2)°C	

References: ^1^Cui *et al.* (2026), ^2^Kuwata *et al.* (2024b), ^3^Kuwata *et al.* (2025), ^4^Chen *et al.* (2021), ^5^Huang *et al.* (2021), ^6^Sildever *et al.* (2019), ^7^Kuwata *et al.* (2023), ^8^Fu *et al.* (2021), ^9^Liu *et al.* (2023), ^10^Chai *et al.* (2026), ^11^Luo *et al.* (2013), ^12^Tao *et al.* (2025), ^13^  Liu et al. (2022), ^14^Sildever *et al.* (2022), ^15^Funaki *et al.* (2025), ^16^Shi *et al.* (2025), ^17^Gu *et al.* (2013), ^18^Takahashi *et al.* (2021), ^19^Potvin *et al.* (2012), ^20^Wang *et al.* (2022), ^21^Wu *et al.* (2024).

### Quantitative PCR

**Fig. 6:**
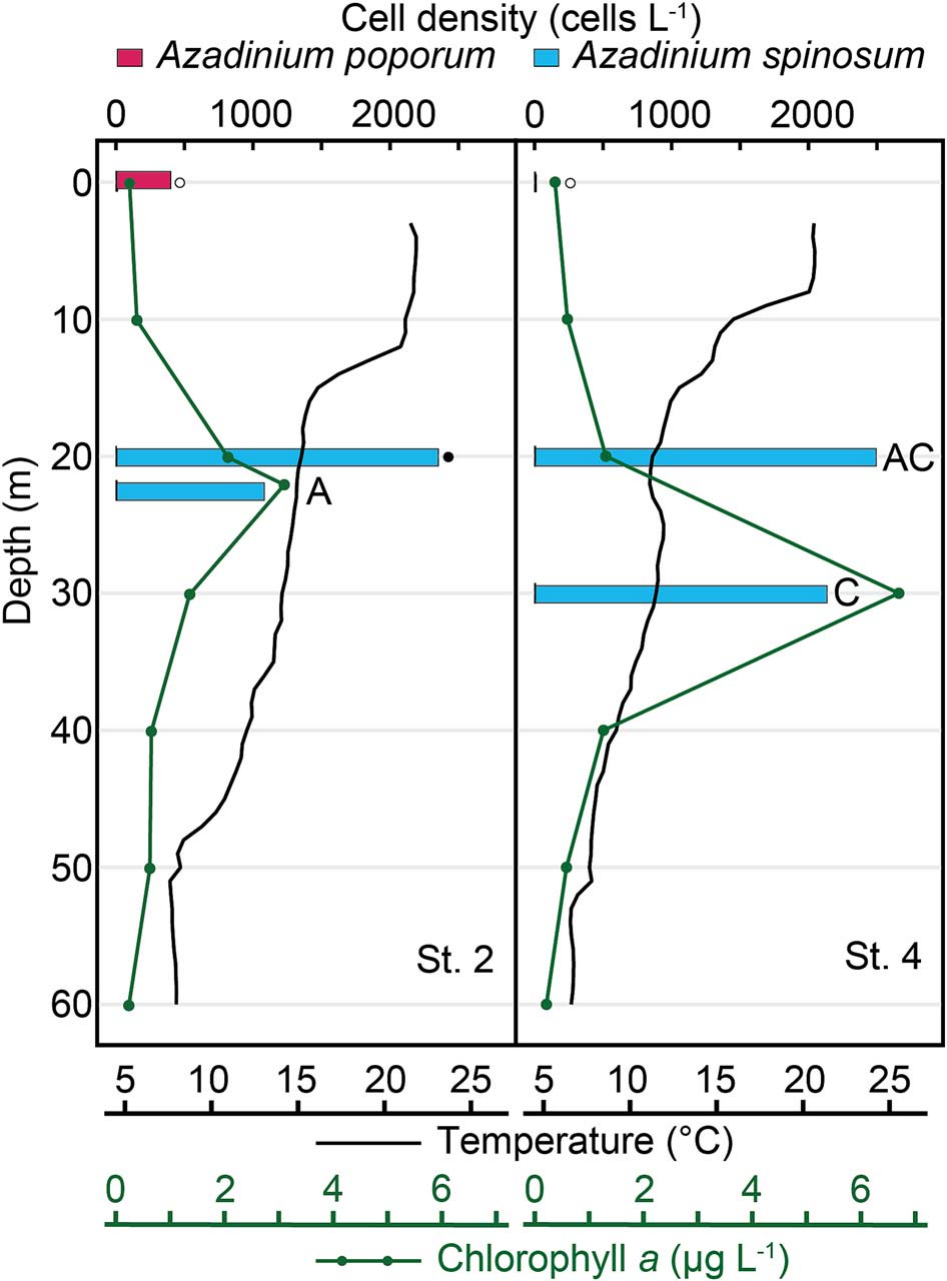
Vertical distribution of cell densities of *Azadinium poporum* (red bars) and *Az. spinosum* (blue bars) estimated by LSU rDNA-based qPCR, off eastern Hokkaido, Japan, in August 2023. Symbols associated with bars indicate the ribotypes detected in the same samples using ITS2-based metabarcoding: Ribotypes of *Az. spinosum* (A, C), not detected (white circles), and no data (black circles). Water temperature (black lines) and concentration of chlorophyll *a* (green lines) are shown.

**Fig. 7:**
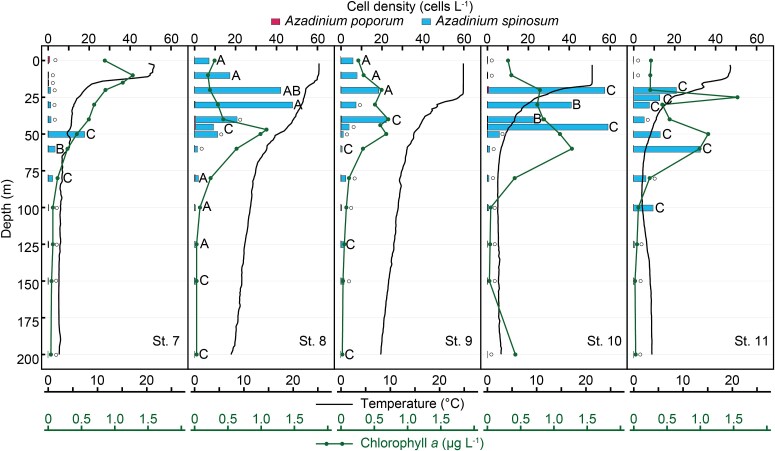
Vertical distribution of cell densities of *Azadinium poporum* (red bars) and *Az. spinosum* (blue bars) estimated by LSU rDNA-based qPCR, off eastern Hokkaido, Japan, in September 2024. Symbols associated with bars indicate the ribotypes detected in the same samples using ITS2-based metabarcoding: Ribotypes of *Az. spinosum* (A, C), and not detected (white circles). Water temperature (black lines) and concentration of chlorophyll *a* (green lines) are shown.

Both *Az*. *poporum* and *Az*. *spinosum* were detected by qPCR (Figs 6, 7). In 2023, *Az*. *poporum* was found at the sea surface layer of St. 2 (394 cells L^−1^) and rarely detected at deeper layers (< 3 cells L^−1^) (Fig. 6). Cell density of *Az*. *spinosum* exceeded 1000 cells L^−1^ in 20–30 m of St. 2 and 4, with the highest density of 2486 cells L^−1^ at 20 m of St. 4. In 2024, *Az*. *spinosum* was mainly detected at 0–60 m depth, with the peak density of 57 cells L^−1^ at St. 10, whereas cell densities of *Az*. *poporum* were very low (< 1 cells L^−1^) with the peak density of 0.7 cells L^−1^ in 0 m of St. 7 and 20 m of St. 10 (Fig. 7). *Azadinium spinosum* was mainly found (> 5 cells L^−1^) in 20–100 m depth of the cold-water (St. 7, 10, 11) and in 0–50 m of the warm-water (St. 8, 9). In the surface seawater samples, *Az*. *spinosum* was detected with the highest density of 80.8 cells L^−1^, while cell densities of *Az*. *poporum* was below 20 cells L^−1^.

### Detection of *Azadinium spinosum* using eDNA metabarcoding and qPCR

*Azadinium spinosum* was detected in 40 samples using metabarcoding and in 88 samples using qPCR (Fig. S6). Among the 40 samples in which the species was detected by metabarcoding, it was not detected in three samples by qPCR. Among the 88 samples positive for the species by qPCR, it was not detected in 51 samples by metabarcoding. There was no positive correlation (*P* > 0.001, R^2^ = 0.06329) between the ASV read numbers (metabarcoding) and cell densities (qPCR), except for higher read numbers of > 300 reads (Fig. S6B), although there was positive correlation with all data (*P* < 0.001, R^2^ = 0.7574) (Fig. S6C).

### Culture strain of *Azadinium spinosum*

A culture strain of *Az. spinosum* (NK0178) isolated in this study was positioned in the clade of *Az*. *spinosum* but was not assigned to any ribotypes in the phylogenetic tree (Fig. 3). Cells were ellipsoid, 11.7–15.7 μm (mean 13.5 ± 0.7 μm, *n* = 15) in length and 7.5–9.6 μm (mean 8.6 ± 0.4 μm, *n* = 15) in width (Fig. S7). The apical pore complex was prominent, and a small antapical spine was visible, but the thecal arrangement was not clearly determined.

## DISCUSSION

### Species diversity of Amphidomataceae

The ITS2-based eDNA metabarcoding revealed the occurrence of 14 amphidomatacean dinoflagellates in the offshore area off eastern Hokkaido, Japan, which includes 11 *Azadinium* and three *Amphidoma* species. Notably, cells of these six species have not been observed previously in the western North Pacific, i.e. two *Amphidoma* (*Am*. *languida* and *Am*. *parvula*) and four *Azadinium* (*Az*. *concinnum*, *Az*. *obesum*, *Az*. *perforatum*, and *Az*. *polongum*). Of these, *Am. languida*, *Am*. *parvula*, *Az. obesum*, *Az*. *perforatum* and *Az. polongum* have so far been detected by eDNA metabarcoding in the western North Pacific (Sildever *et al.,* 2019; Chen *et al.,* 2021; Huang *et al.,* 2021; Liu *et al.,* 2023; Shi *et al.,* 2025; Cui *et al.,* 2026), and a germinated cell of *Az. obesum* was isolated from the seabed sediment in the United States, eastern North Pacific (Kim *et al.,* 2017). Regarding the previous reports of *Am. languida*, however, eDNA in the Okhotsk Sea was identified based on SSU rDNA (V7–V9), which lacks sufficient resolution to differentiate *Am. languida* and *Am. fulgens* precisely, and in fact, the sequences initially reported as *Am*. *languida* detected from the Bohai Sea and East China Sea were later revealed to represent *Am. fulgens* (Sildever *et al.,* 2019; Chen *et al.,* 2021; Huang *et al.,* 2021; Kuwata *et al.,* 2024b). In this study, the ASV 2822 was found to belong to the clade of AZA-producing *Am. languida* strains from Ireland and Mexico (Tillmann *et al.,* 2012a; Kuwata *et al.,* 2024a). Detection of *Az. concinnum* represent the first records of these species in the Pacific. Conversely, six *Azadinium* species previously reported in the western North Pacific were not found in this study. Cells of *Az*. *cuneatum*, *Az. fusiforme*, *Az. inconspicuum*, and *Az*. *zhuanum* have been found in the coasts of the East China Sea, China; Mutsu Bay, Sagami Bay, and Seto Inland Sea, Japan; and Namhae, Korea (Luo *et al.,* 2013; Takahashi *et al.,* 2021; Kuwata *et al.,* 2025; Cui *et al.,* 2026; Tillmann *et al.,* 2026). Additionally, *Az. galwayense* and *Az. perfusorium* have been detected from eDNAs in the Taiwan Strait, East China Sea, and Yellow Sea (Liu *et al.,* 2023; Shi *et al.,* 2025; Tao *et al.,* 2025; Cui *et al.,* 2026). Given that these localities represent coastal regions influenced by warm currents, it is likely that these *Azadinium* species are rare in the offshore or cold-water regions examined in this study.

### Genetic diversity of Amphidomataceae

A high genetic diversity of the Amphidomataceae was discovered off the Pacific coast of eastern Hokkaido, Japan. This includes ASVs belonging to the 14 species mentioned earlier and others that are not clearly assigned to the described species. Many of the unidentified ASVs were phylogenetically positioned close to known species, e.g. *Az. anteroporum*, *Az. concinnum*, *Az. dexteroporum*, *Az.* cf. *galwayense*, *Az.* cf. *inconspicuum*, *Az. perfusorium*, *Az. polongum*, *Az.* cf. *spinosum*, *Az. trinitatum*, *Am. languida*, and *Am. pontica*. Some of the sequences probably belong to the related amphidomatacean species, but it was difficult to judge the species affiliation without their morphological information.

Among the unidentified ASVs, four clades emerged as distinct lineages and were designated as *Azadinium* sp. 1–4; they are likely undescribed species, or species of the family Amphidomataceae known only from morphology. *Azadinium* sp. 1 was positioned within the clade of *Azadinium* species characterized by the ventral pore located on the left side of the Po plate, i.e. *Az. anteroporum*, *Az. cuneatum*, *Az. dalianense*, *Az. fusiforme*, *Az. obesum*, *Az. poporum*, *Az. spinosum*, and *Az. trinitatum* (Tillmann *et al.,* 2009, 2010, 2011, 2014, 2026; Luo *et al.,* 2013; Kuwata *et al.,* 2023). The phylogeny of *Azadinium* sp. 2 and sp. 3, which was positioned paraphyly to the aforementioned clade, was similar to that reported from the Taiwan Strait as *Azadinium* sp. 1, sp. 6, and sp. 7 (Liu *et al.,* 2023). In this study, *Azadinium* sp. 2 and sp. 3 were detected in the warm-water. *Azadinium* sp. 4 was closely positioned at the clade that included *Az. dexteroporum*, *Az. inconspicuum*, *Az. galwayense*, and *Az. perfusorium* (Percopo *et al.,* 2013; Salas *et al.,* 2021; Kuwata *et al.,* 2025). It was found in the cold-water in this study, and the related clade was not reported from the Taiwan Strait (Liu *et al.,* 2023). Strains with the relevant sequences are anticipated to be generated utilizing the environment data obtained from eDNA metabarcoding.

### Amphidomataceans common in pelagic water off Hokkaido, Japan

Off the coast of Hokkaido, Japan, lies the region influenced by the cold (Oyashio) and warm (Kuroshio) water currents. During the 2024 cruise, seawater samples were categorized into the cold-water (St. 7, 10, 11) and warm-water (St. 8, 9) based on their temperature. The vertical profiles in the cold-water stations showed higher temperatures at the surface and lower temperatures at deeper layers below 50 m, suggesting that the cold-water masses originated from subarctic waters and were subsequently warmed at the surface. In contrast, the higher temperature profiles in the warm-water stations indicate that this water mass originated from Kuroshio–Oyashio mixed water and was also warmed. To gain a better understanding of the distribution and ecological traits of amphidomataceans, the originating water mass and distribution temperatures were estimated for abundantly occurring amphidomataceans at species, intraspecific group (ribotype), and sequence (ASV) levels. In the 2024 cruise, *Am. fulgens* and *Az. dexteroporum* were found in many warm-water samples, while *Az. dalianense* and *Az. polongum* were found in the cold-water samples, and *Az. spinosum* was present in both water masses. Regarding the vertical distribution, it was challenging to discern due to the intricate structure of seawater. In the surface layers (0–10 m), amphidomataceans which were mainly found in the warm-water (e.g. *Am. fulgens* and *Az. dexteroporum*) were present, while they in the cold-water (e.g. *Az. dalianense* and *Az. polongum*) were absent. Moreover, numerous species which were identified in surface waters collected using the automated filtration system were detected in the warm-water samples simultaneously. It remains unclear whether the distribution reflects the habitat preferences of the species or merely results from transport with surface waters originating from warm-water masses.

*Amphidoma fulgens*, a non-toxigenic species related to the toxigenic species *Am. languida*, has so far been reported from the coasts of Japan, Malaysia, and the Philippines (Kuwata *et al.,* 2024b, 2025). The eDNA sequences reported from the Chinese coasts as *Am. languida* have recently been shown to represent *Am. fulgens* (Chen *et al.,* 2021; Huang *et al.,* 2021; Kuwata *et al.,* 2024b), suggesting that the species is widely distributed in the temperate and sub-tropical coastal waters in the western North Pacific. In this study, this species was mostly detected in the warm-water (mean 14.7°C), which also suggests its distribution mainly in warm environments, and additionally, indicates that it may commonly inhabit offshore water. On the other hand, the related toxigenic species *Am. languida* exists in this region, as mentioned above, but was rarely detected in this study. Twenty-five sequences were assigned to the clade containing *Am*. *fulgens* and *Am*. *languida*, yet the internal clades could not be clearly resolved, indicating high genetic diversity within the clade.

*Azadinium dalianense* was primarily found in the cold-water (mean 5.6°C). This non-toxigenic species have been found from the coastal sediments of China and United States in the North Pacific (Luo *et al.,* 2013; Kim *et al.,* 2017; Tao *et al.,* 2025; Chai *et al.,* 2026; Cui *et al.,* 2026), and the motile cells have also been found in the Atlantic (Denmark, France, Norway) and Pacific (Japan, New Zealand) (Luo *et al.,* 2013, 2017; Tillmann *et al.,* 2018b, 2019; Wietkamp *et al.,* 2019b; Balci *et al.,* 2023; Kuwata *et al.,* 2025). Among the three ribotypes (A, C, E) found in this study, the ribotype C (mean 10.1°C) and ribotype E (mean 4.7°C) were detected in the five stations of the 2024 cruise. Since a culture strain of ribotype C was isolated from the Pacific seawater off Hokkaido, Japan (12–13°C), and germinated cells were obtained from coastal sediments of the United States (Kim *et al.,* 2017; Kuwata *et al.,* 2025), this ribotype may be distributed near the coasts.

*Azadinium dexteroporum* was found mainly in the warm-waters (mean 15.8°C) in 2024. In the Pacific, only one strain of *Az. dexteroporum* was isolated from pelagic water ca. 600 km far from the main island of Japan (Kuwata *et al.,* 2025); therefore, it may be distributed in the offshore region of the western North Pacific. Interestingly, most of the DNA sequences phylogenetically related to *Az. dexteroporum*, such as *Az.* cf. *galwayense* and *Az.* cf. *inconspicuum*, were found in the warm-water, suggesting that these members are distributed mainly in warm environments in the western North Pacific. An exception is a DNA sequence (ASV 862) belonging to the ribotype B of *Az. dexteroporum*, which was detected mainly in the cold-water. Given that the two existing ribotype B strains were both isolated from the cold North Atlantic waters (Irminger Sea and Labrador Sea) (Tillmann *et al.,* 2015, 2020), the ribotype B was possible to distribute in cold environments. *Azadinium dexteroporum* is originally described as an AZA-producing species with the strain isolated from Naples, Italy (ribotype A); however, none of the AZA analogs has been detected in the strains of the species isolated later from the Irminger Sea (ribotype B) and Japan (ribotype C) (Percopo *et al.,* 2013; Tillmann *et al.,* 2015; Kuwata *et al.,* 2025). In this study, none of the thirteen detected DNA sequences were assigned to the toxigenic ribotype A of *Az. dexteroporum*.

*Azadinium polongum* was found mainly in the cold-water (mean 6.5°C) in the 2024 cruise, including the same ASV which was also detected in the warm-water (18.4°C). Although cells of *Az. polongum* were not found in the western North Pacific, its eDNA has been detected using metabarcoding in water and sediment samples in China (Liu *et al.,* 2023; Shi *et al.,* 2025; Tao *et al.,* 2025; Cui *et al.,* 2026). This species has rarely been reported from this region, making its abundant detection in this study unexpected. However, this predominant occurrence of *Az. polongum* in the non-coastal area is congruent with the previous observations in other regions that the species is originally found from offshore near the Shetland Islands, and its algal bloom formed off the coast of Peru (Tillmann *et al.,* 2012b, 2017).

### Metabarcoding and qPCR detections of two toxigenic *Azadinium*

*Azadinium poporum* was rarely found during the 2023 cruise, with the ribotypes A2, B, and C1, and was not found in 2024. In the Amphidomataceae, this is the most frequently reported species in coastal waters in Japan, with the ribotypes A1, A2, B, and C1 (Takahashi *et al.,* 2021; Kuwata *et al.,* 2025). In contrast to its wide distribution across the coastal environment, which is probably related to the ability to form cysts (Gu *et al.,* 2013), these eDNA detections suggest that it is rare in the pelagic waters in this region. Species-specific qPCR detection also shows the infrequent occurrence of *Az. poporum*, although the sporadic occurrences with the cell density of 394 cells L^−1^ in the surface water during the 2023 cruise.

*Azadinium spinosum* was found both in warm- and cold-waters, with a wide temperature range of 1.6–25.6°C. It suggests that the toxigenic *Az. spinosum* was distributed commonly in the offshore region, although it has rarely been found in the Pacific to date, with only a single isolate of the ribotype A obtained from the Seto Inland Sea, Japan (Kuwata *et al.,* 2025). In this study, three distinct ribotypes of *Az. spinosum* were found in different temperature conditions; the ribotype A in the warm-water (mean 19.9°C), ribotype B in both the warm-water (22.6°C) and cold-water (mean 5.8°C), and ribotype C mainly in the cold-water (mean 5.8°C). The varying distribution temperature among *Az. spinosum* ribotypes align with previous findings in the western North Pacific; only the ribotypes A and B have been detected in the Gulf of Thailand (water temperature exceeds 26°C) and in the Taiwan Strait (Fu *et al.,* 2021; Liu *et al.,* 2023). Several strains of *Az. spinosum* ribotype C have been shown to lack azaspiracid production (Tillmann *et al.,* 2019); however, these strains were exclusively isolated from the Argentinean shelf, where recurrent blooms of amphidomataceans have been reported (Tillmann *et al.,* 2019; Guinder *et al.,* 2025). Consequently, the AZA-production potential of the ribotype inhabiting this region is uncertain. We therefore explored *Azadinium* cells off the Pacific coast of Hokkaido and could isolate one strain (NK0178) of *Az. spinosum*, but this strain was lost before the AZA analysis, unfortunately.

The discovery that *Az. spinosum* was commonly distributed in the offshore region of the Pacific, with relatively high cell densities (maximum 2486 cells L^−1^), is noteworthy. This suggests the different distribution between two important AZA-producing species. In contrast to *Az. poporum*, which is widely distributed across the coastal waters (Takahashi *et al.,* 2021; Kuwata *et al.,* 2025), *Az. spinosum* predominantly occurs in the offshore waters of the western North Pacific.

### Technical challenges of eDNA to detect Amphidomataceae

DNA detections of *Az*. *spinosum* using two different methods, ITS2-based eDNA metabarcoding and LSU rDNA-based qPCR, indicated a slightly higher detectability of the qPCR than that of eDNA metabarcoding. However, there was no clear correlation between the sequence (ASV) read numbers detected by metabarcoding and cell densities estimated by qPCR in most samples with low values (< 300 ASV reads; < 100 cells L^−1^). This discrepancy may be attributed to the variability in ASV read numbers depending on the cell and species numbers of other dinoflagellates, as the primer set is designed to be universal for all dinoflagellates (Fu *et al.,* 2021). In addition, a PCR bias of copy numbers which caused by two-step PCR in eDNA metabarcoding was possible to weaken the correlation between two methods. Considering that *Az. spinosum* comprises both toxigenic and non-toxigenic ribotypes, a combination of species-specific qPCR and ribotype-discernible metabarcoding can provide useful information for harmful algal bloom monitoring.

In this study, eDNA metabarcoding using ITS2 region allowed for the detection and identification of the Amphidomataceae at a fine resolution, except for species known only from morphology (e.g. Tillmann *et al.,* 2016; Tillmann, 2018). While it revealed the genetic diversity within the family, it posed challenges in distinguishing genetic polymorphisms of ITS2 due to multiple copies of rDNA sequences. This study identified a high species diversity of the Amphidomataceae, including eDNA sequences related to described species, although the identities of several sequences were not definitively established. This underscores the significance of available reference DNA sequences, characterized also with the morphology and toxin productivity, for molecular detection of toxigenic microalgae in environmental DNA.

## CONCLUSIONS

ITS2-based eDNA metabarcoding study conducted in 2023 and 2024 revealed a remarkable genetic and species diversity within the dinoflagellate family Amphidomataceae in offshore water off the Pacific coast of Hokkaido, Japan. It identified a total of 14 species, including 11 *Azadinium* species (*Az. anteroporum*, *Az. caudatum*, *Az. concinnum*, *Az. dalianense*, *Az. dexteroporum*, *Az. obesum*, *Az. perforatum*, *Az. polongum*, *Az. poporum*, *Az. spinosum*, and *Az. trinitatum*) and three *Amphidoma* species (*Am. parvula*, *Am. fulgens*, and *Am. languida*). Notably, *Az. concinnum* was new record in the Pacific Ocean, with an unambiguous molecular identification of the toxigenic *Am. languida* in the western North Pacific. Predominant occurrence of *Az. spinosum* (ribotype C) was also discovered in the Pacific for the first time. The ITS phylogeny also revealed four unidentified sequences of *Azadinium* sp. 1–4, which may represent four undescribed *Azadinium* species. eDNA detections in 65 seawater samples in the 2024 cruise showed that the possibility for different temperature-associated distribution to be observed at the species and intraspecific level; *Am. fulgens*, *Az. dexteroporum*, and *Az. spinosum* (ribotype A) were found in the warm-water (warming Kuroshio-Oyashio mixed water), whereas *Az. dalianense*, *Az. polongum*, and *Az. spinosum* (ribotype C) were detected in the cold-water (representing surface-warmed subarctic water). Species-specific qPCR analysis of two toxigenic *Azadinium* species revealed the predominant distribution of *Az. spinosum* in the pelagic western North Pacific for the first time, with relatively high cell densities (maximum 2486 cells L^−1^). In contrast, *Az. poporum*, which has been commonly detected in coastal waters, was rare in pelagic waters.

## Supplementary Material

Supplementary_materials_fbag029

## Data Availability

The data used in this study are available in Figshare at: 10.6084/m9.figshare.31148959.

## References

[ref1] Adams, N. G., Tillmann, U. and Trainer, V. L. (2020) Temporal and spatial distribution of *Azadinium* species in the inland and coastal waters of the Pacific northwest in 2014–2018. Harmful Algae, 98, 101874. 10.1016/j.hal.2020.101874. 33129464

[ref2] Balci, M., Rhodes, L. L., Nishimura, T., Murray, J. S., Harwood, D. T., MacKenzie, A. L., Bui, T., Moisan, C. et al. (2023) Molecular detection and distribution of the genera *Amphidoma* and *Azadinium* (Amphidomataceae, Dinophyceae) in the coastal waters of Aotearoa/New Zealand. N. Z. J. Mar. Freshw. Res., 57, 47–62. 10.1080/00288330.2021.1953083.

[ref3] Callahan, B. J., McMurdie, P. J. and Holmes, S. P. (2017) Exact sequence variants should replace operational taxonomic units in marker-gene data analysis. ISME J., 11, 2639–2643. 10.1038/ismej.2017.119. 28731476 PMC5702726

[ref4] Callahan, B. J., McMurdie, P. J., Rosen, M. J., Han, A. W., Johnson, A. J. A. and Holmes, S. P. (2016) DADA2: high-resolution sample inference from Illumina amplicon data. Nat. Methods, 13, 581–583. 10.1038/nmeth.3869. 27214047 PMC4927377

[ref5] Chai, Z., Tao, Z., Yue, C., Li, R., Liu, Y., Shi, S., Yang, Y., Sun, K. et al. (2026) Shifts of dinoflagellate cyst assemblages over the past 100 years indicate the history of harmful algae blooms, eutrophication and possible alien genotype invasion around the Shandong Peninsula, China. Mar. Pollut. Bull., 223, 119064. 10.1016/j.marpolbul.2025.119064. 41317645

[ref6] Chen, Y., Xu, Q., Gibson, K. and Chen, N. (2021) Metabarcoding dissection of harmful algal bloom species in the East China Sea off southern Zhejiang Province in late spring. Mar. Pollut. Bull., 169, 112586. 10.1016/j.marpolbul.2021.112586. 34116370

[ref7] Cui, L., Hu, W., Tillmann, U., Iwataki, M., Chen, Y., Kang, J., Hii, K. S., Mohamed, H. F. et al. (2026) Unlocking species and subspecies diversity of harmful microalgae using HiFi long-read metabarcoding: insights from Amphidomataceae (Dinophyceae) in the China Sea. Harmful Algae, 155, 103103. 10.1016/j.hal.2026.103103. 42025387

[ref8] Fu, Z., Piumsomboon, A., Punnarak, P., Uttayarnmanee, P., Leaw, C. P., Lim, P. T., Wang, A. and Gu, H. (2021) Diversity and distribution of harmful microalgae in the Gulf of Thailand assessed by DNA metabarcoding. Harmful Algae, 106, 102063. 10.1016/j.hal.2021.102063. 34154784

[ref9] Funaki, H., Gaonkar, C. C., Nishimura, T., Tanaka, K., Kamimura, K., Kaji, T., Nagasaki, K. and Adachi, M. (2025) Vertical distribution of harmful algae in the sediment of Uranouchi Inlet by metabarcoding. Front. Protistol., 3, 1612811. 10.3389/frpro.2025.1612811.

[ref10] Gu, H., Luo, Z., Krock, B., Witt, M. and Tillmann, U. (2013) Morphology, phylogeny and azaspiracid profile of *Azadinium poporum* (Dinophyceae) from the China Sea. Harmful Algae, 21-22, 64–75. 10.1016/j.hal.2012.11.009. 28073547

[ref11] Guinder, V. A., Tillmann, U., Rivarossa, M., Ferronato, C., Ramírez, F. J., Krock, B., Gu, H. and Saraceno, M. (2025) Extraordinary bloom of toxin-producing phytoplankton enhanced by strong retention of the offshore Patagonian shelf. Biogeosciences, 22, 3397–3428. 10.5194/bg-22-3397-2025.

[ref12] Huang, H., Xu, Q., Gibson, K., Chen, Y. and Chen, N. (2021) Molecular characterization of harmful algal blooms in the Bohai Sea using metabarcoding analysis. Harmful Algae, 106, 102066. 10.1016/j.hal.2021.102066. 34154783

[ref13] Iwataki, M., Lum, W. M., Kuwata, K., Takahashi, K., Arima, D., Kuribayashi, T., Kosaka, Y., Hasegawa, N. et al. (2022) Morphological variation and phylogeny of *Karenia selliformis* (Gymnodiniales, Dinophyceae) in an intensive cold water algal bloom in eastern Hokkaido, Japan. Harmful Algae, 114, 102204. 10.1016/j.hal.2022.102204. 35550287

[ref14] Katoh, K., Rozewicki, J. and Yamada, K. D. (2019) MAFFT online service: multiple sequence alignment, interactive sequence choice and visualization. Brief. Bioinform., 20, 1160–1166. 10.1093/bib/bbx108. 28968734 PMC6781576

[ref15] Kim, J. H., Tillmann, U., Adams, N. G., Krock, B., Stutts, W. L., Deeds, J. R., Han, M. S. and Trainer, V. L. (2017) Identification of *Azadinium* species and a new azaspiracid from *Azadinium poporum* in Puget Sound, Washington state, USA. Harmful Algae, 68, 152–167. 10.1016/j.hal.2017.08.004. 28962976

[ref16] Krock, B., Tillmann, U., Tebben, J., Trefault, N. and Gu, H. (2019) Two novel azaspiracids from *Azadinium poporum*, and a comprehensive compilation of azaspiracids produced by Amphidomataceae, (Dinophyceae). Harmful Algae, 82, 1–8. 10.1016/j.hal.2018.12.005. 30928006

[ref17] Krock, B., Tillmann, U., Voβ, D., Koch, B. P., Salas, R., Witt, M., Potvin, E. and Jeong, H. J. (2012) New azaspiracid in Amphidomataceae (Dinophyceae). Toxicon, 60, 830–839. 10.1016/j.toxicon.2012.05.007. 22643573

[ref18] Krock, B., Tillmann, U., Witt, M. and Gu, H. (2014) Azaspiracid variability of *Azadinium poporum* (Dinophyceae) from the China Sea. Harmful Algae, 36, 22–28. 10.1016/j.hal.2014.04.012.

[ref19] Kumar, S., Stecher, G., Li, M., Knyaz, C. and Tamura, K. (2018) MEGA X: molecular evolutionary genetics analysis across computing platforms. Mol. Biol. Evol., 35, 1547–1549. 10.1093/molbev/msy096. 29722887 PMC5967553

[ref20] Kuwata, K., Hernández-Becerril, D. U., Lum, W. M., Takahashi, K., Benico, G., Uchida, H., Ozawa, M., Suzuki, T. et al. (2024a) First report of toxigenic *Amphidoma languida* (Amphidomataceae, Dinophyceae) from the Pacific, with reference to intracellular ultrastructure and azaspiracid compounds. Phycol. Res., 72, 266–278. 10.1111/pre.12567.

[ref21] Kuwata, K., Lum, W. M., Takahashi, K., Benico, G., Ozawa, M., Uchida, H., Numano, S., Watanabe, R. et al. (2025) Diversity of amphidomatacean dinoflagellates in Japan, with a description of *Azadinium inconspicuum* sp. nov. and azaspiracid components in *Azadinium poporum* ribotypes. Harmful Algae, 150, 102969. 10.1016/j.hal.2025.102969. 41241523

[ref22] Kuwata, K., Lum, W. M., Takahashi, K., Benico, G., Takahashi, K., Lim, P. T., Leaw, C. P., Uchida, H. et al. (2024b) Phylogeny and ultrastructure of a non-toxigenic dinoflagellate *Amphidoma fulgens* sp. nov. (Amphidomataceae, Dinophyceae), with a wide distribution across Asian Pacific. Harmful Algae, 138, 102701. 10.1016/j.hal.2024.102701. 39244236

[ref23] Kuwata, K., Lum, W. M., Takahashi, K., Benico, G., Uchida, H., Ozawa, M., Matsushima, R., Watanabe, R. et al. (2023) A new small thecate dinoflagellate *Azadinium anteroporum sp*. *nov*. (Amphidomataceae, Dinophyceae) isolated from the Asian Pacific. Phycologia, 62, 303–314. 10.1080/00318884.2023.2204681.

[ref24] Larsson, A. (2014) AliView: a fast and lightweight alignment viewer and editor for large dataset. Bioinformatics, 30, 3276–3278. 10.1093/bioinformatics/btu531. 25095880 PMC4221126

[ref25] Liu, M., Tillmann, U., Ding, G., Wang, A. and Gu, H. (2023) Metabarcoding revealed a high diversity of Amphidomataceae (Dinophyceae) and the seasonal distribution of their toxigenic species in the Taiwan Strait. Harmful Algae, 124, 102404. 10.1016/j.hal.2023.102404. 37164557

[ref25a] Liu, S., Cui, Z., Zhao, Y. and Chen, N. (2022) Composition and spatial-temporal dynamics of phytoplankton community shaped by environmental selection and interactions in the Jiaozhou Bay. Water Res., 218, 118488. 10.1016/j.watres.2022.118488. 35489150

[ref26] Luo, Z., Gu, H., Krock, B. and Tillmann, U. (2013) *Azadinium dalianense*, a new dinoflagellate species from the Yellow Sea, China. Phycologia, 52, 625–636. 10.2216/13-178.1.

[ref27] Luo, Z., Krock, B., Mertens, K. N., Nézan, E., Chomérat, N., Bilien, G., Tillmann, U. and Gu, H. (2017) Adding new pieces to the *Azadinium* (Dinophyceae) diversity and biogeography puzzle: non-toxigenic *Azadinium zhuanum* sp. nov. from China, toxigenic *A*. *poporum* from the Mediterranean, and a non-toxigenic *A*. *dalianense* from the French Atlantic. Harmful Algae, 66, 65–78. 10.1016/j.hal.2017.05.001. 28602255

[ref28] Luo, Z., Krock, B., Mertens, K. N., Price, A. M., Turner, R. E., Rabalais, N. N. and Gu, H. (2016) Morphology, molecular phylogeny and azaspiracid profile of *Azadinium poporum* (Dinophyceae) from the Gulf of Mexico. Harmful Algae, 55, 56–65. 10.1016/j.hal.2016.02.006. 28073547

[ref29] Martin, M. (2011) Cutadapt removes adapter sequences from eDNA sequencing reads. EMBnet. journal, 17, 10–12. 10.14806/ej.17.1.200.

[ref30] Nézan, E., Tillmann, U., Bilien, G., Boulben, S., Chèze, K., Zentz, F., Salas, R. and Chomérat, N. (2012) Taxonomic revision of the dinoflagellate *Amphidoma caudata*: transfer to the genus *Azadinium* (Dinophyceae) and proposal of two varieties, based on morphological and molecular phylogenetic analyses. J. Phycol., 48, 925–939. 10.1111/j.1529-8817.2012.01159.x. 27009003

[ref31] Nylander, J. A. A. (2004) MrModeltest v2, Evolutionary Biology Centre, Uppsala University, Program distributed by the author.

[ref32] Okazaki, Y., Nguyen, T. T., Nishihara, A., Endo, H., Ogata, H., Nakano, S. and Tamaki, H. (2023) A fast and easy method to co-extract DNA and RNA from an environmental microbial sample. Microbes Environ., 38, ME22102. 10.1264/jsme2.ME22102. PMC10037101 36928278

[ref33] Ozawa, M., Uchida, H., Numano, S., Watanabe, R., Matsushima, R., Oikawa, H., Takahashi, K., Iwataki, M. et al. (2025) New azaspiracid analogues produced by *Azadinium spinosum* isolated from Japanese coastal waters. In Imai, I., Matsushima, R., Nagai, S., Nishitani, G., Sakamoto, S. and Suzuki, T. (eds.), Proceedings of 20th International Conference on Harmful Algae. International Society for the Study of Harmful Algae, Hiroshima, pp. 89–92. 10.15027/0002041274.

[ref34] Ozawa, M., Uchida, H., Watanabe, R., Matsushima, R., Oikawa, H., Takahashi, K., Iwataki, M. and Suzuki, T. (2021) Complex profiles of azaspiracid analogues in two culture strains of *Azadinium poporum* (Amphidomataceae, Dinophyceae) isolated from Japanese coastal waters determined by LC-MS/MS. Toxicon, 199, 145–155. 10.1016/j.toxicon.2021.06.010. 34166679

[ref35] Ozawa, M., Uchida, H., Watanabe, R., Matsushima, R., Oikawa, H., Takahashi, K., Iwataki, M. and Suzuki, T. (2023) Azaspiracid accumulation in Japanese coastal bivalves and ascidians fed with *Azadinium poporum* producing azaspiracid-2 as the dominant toxin component. Toxicon, 226, 107069. 10.1016/j.toxicon.2023.107069. 36871920

[ref36] Ozawa, M., Uchida, H., Watanabe, R., Numano, S., Matsushima, R., Oikawa, H., Takahashi, K., Lum, W. M. et al. (2024) New azaspiracid analogues detected as bi-charged ions in *Azadinium poporum* (Amphidomataceae, Dinophyceae) isolated from Japanese coastal waters. J. Chromatogr. B, 1236, 124065. 10.1016/j.jchromb.2024.124065. 38460449

[ref37] Percopo, I., Siano, R., Rossi, R., Soprano, V., Sarno, D. and Zingone, A. (2013) A new potentially toxic *Azadinium* species (Dinophyceae) from the Mediterranean Sea, *A. dexteroporum* sp. nov. J. Phycol., 49, 950–966. 10.1111/jpy.12104. 27007318

[ref38] Potvin, É., Jeong, H. J., Kang, N. S., Tillmann, U. and Krock, B. (2012) First report of the photosynthetic dinoflagellate genus *Azadinium* in the Pacific Ocean: morphology and molecular characterization of *Azadinium* cf. *poporum*. J. Eukaryot. Microbiol., 59, 145–156. 10.1111/j.1550-7408.2011.00600.x. 22188605

[ref39] R Core Team (2023) R: A Language and Environment for Statistical Computing, R Foundation for Statistical Computing, Vienna, Austria, https://www.R-project.org/.

[ref40] Ronquist, F., Teslenko, M., van der Mark, P., Ayres, D. L., Darling, A., Höhna, S., Larget, B., Liu, L. et al. (2012) MrBayes 3.2: efficient Bayesian phylogenetic inference and model choice across a large space. Syst. Biol., 61, 539–542. 10.1093/sysbio/sys029. 22357727 PMC3329765

[ref41] Salas, R., Tillmann, U., Gu, H., Wietkamp, S., Krock, B. and Clarke, D. (2021) Morphological and molecular characterization of multiple new *Azadinium* strains revealed a high diversity of non-toxigenic species of Amphidomataceae (Dinophyceae) including two new species in Irish waters, north East Atlantic. Phycol. Res., 69, 88–115. 10.1111/pre.12448.

[ref42] Sato, S., Nishimura, T., Uehara, K., Sakanari, H., Tawong, W., Hariganeya, N., Smith, K., Rhodes, L. et al. (2011) Phylogeography of *Ostreopsis* along West Pacific coast, with special reference to a novel clade from Japan. PLoS One, 6, e27983. 10.1371/journal.pone.0027983. 22164222 PMC3229513

[ref43] Scholin, C. A., Herzog, M., Sogin, M. and Anderson, D. M. (1994) Identification of group- and strain-specific genetic markers for globally distributed *Alexandrium* (Dinophyceae). II. Sequence analysis of a fragment of the LSU rRNA gene. J. Phycol., 30, 999–1011. 10.1111/j.0022-3646.1994.00999.x.

[ref44] Shi, S., Tao, Z., Yang, W., Li, F., Wei, B., Yue, C., Pan, S., Deng, Y. et al. (2025) A newly developed Germlings Harvester (GEHA) in combination with metabarcoding analysis detected numerous plankton species, particularly HABs-causing species, from in-situ germination of resting stage cells. Harmful Algae, 150, 103002. 10.1016/j.hal.2025.103002. 41241549

[ref45] Sildever, S., Kawakami, Y., Kanno, N., Kasai, H., Shiomoto, A., Katakura, S. and Nagai, S. (2019) Toxic HAB species from the Sea of Okhotsk detected by a metagenetic approach, seasonality and environmental drivers. Harmful Algae, 87, 101631. 10.1016/j.hal.2019.101631. 31349888

[ref46] Sildever, S., Nishi, N., Inaba, N., Asakura, T., Kikuchi, J., Asano, Y., Kobayashi, T., Gojobori et al. (2022) Monitoring harmful microalgal species and their appearance in Tokyo Bay, Japan, using metabarcoding. Metabarcoding Metagenom., 6, 261–280. 10.3897/mbmg.6.79471.

[ref47] Smith, K. F., Rhodes, L., Harwood, D. T., Adamson, J., Moisan, C., Munday, R. and Tillmann, U. (2016) Detection of *Azadinium poporum* in New Zealand: the use of molecular tools to assist with species isolations. J. Appl. Phycol., 28, 1125–1132. 10.1007/s10811-015-0667-5.

[ref48] Stecher, G., Tamura, K. and Kumar, S. (2020) Molecular evolutionary genetics analysis (MEGA) for macOS. Mol. Biol. Evol., 37, 1237–1239. 10.1093/molbev/msz312. 31904846 PMC7086165

[ref49] Swofford, D. L. (2003) PAUP*. Phylogenetic analysis using parsimony (*and other methods). Version 4. Sinauer associates, Massachusetts.

[ref50] Takahashi, K., Lum, W. M., Benico, G., Uchida, H., Ozawa, M., Oikawa, H., Suzuki, T., Nguyen, N. V. et al. (2021) Toxigenic strains of *Azadinium poporum* (Amphidomataceae, Dinophyceae) from Japan and Vietnam, with first reports of *A*. *poporum* (ribotype A) and *A*. *trinitatum* in Asian Pacific. Phycol. Res., 69, 175–187. 10.1111/pre.12455.

[ref51] Tao, Z., Liu, Y., Liu, X., Yue, C., Song, X., Hu, Z., Shi, S., Li, R. et al. (2025) Single-cyst morpho-molecular identification detected an unexpected high species diversity of dinoflagellate resting cysts from the coastal seas of China. Harmful Algae, 149, 102941. 10.1016/j.hal.2025.102941. 40935529

[ref52] Tillmann, U. (2018) Electron microscopy of a 1991 spring plankton sample from the Argentinean shelf reveals the presence of four new species of the Amphidomataceae (Dinophyceae). Phycol. Res., 66, 269–290. 10.1111/pre.12225.

[ref53] Tillmann, U. and Akselman, R. (2016) Revisiting the 1991 algal bloom in shelf waters off Argentina: *Azadinium luciferelloides* sp. nov. (Amphidomataceae, Dinophyceae) as the causative species in a diverse community of other amphidomataceans. Phycol. Res., 64, 160–175. 10.1111/pre.12133.

[ref54] Tillmann, U., Borel, M. R., Barrera, F., Lara, R., Krock, B., Almandoz, G. O., Witt, M. and Trefault, N. (2016) *Azadinium poporum* from the argentine continental shelf, Southwestern Atlantic, produces azaspiracid-2 and azaspiracid-2 phosphate. Harmful Algae, 51, 40–55. 10.1016/j.hal.2015.11.001. 28003061

[ref55] Tillmann, U., Dzhembekova, N., Vlas, O., Krock, B., Boicenco, L. and Dursun, F. (2025) Diversity of Amphidomataceae (Dinophyceae) in the Black Sea, including description of *Amphidoma pontica* sp. nov. Phycol. Res., 73, 225–248. 10.1111/pre.70001

[ref56] Tillmann, U., Edvardsen, B., Krock, B., Smith, K. F., Paterson, R. F. and Voß, D. (2018b) Diversity, distribution, and azaspiracids of Amphidomataceae (Dinophyceae) along the Norwegian coast. Harmful Algae, 80, 15–34. 10.1016/j.hal.2018.08.011. 30502808

[ref57] Tillmann, U., Elbrächter, M., John, U. and Krock, B. (2011) A new non-toxic species in the dinoflagellate genus *Azadinium*: *A*. *poporum* sp. nov. Eur. J. Phycol., 46, 74–87. 10.1080/09670262.2011.556753.

[ref58] Tillmann, U., Elbrächter, M., John, U., Krock, B. and Cembella, A. (2010) *Azadinium obesum* (Dinophyceae), a new nontoxic species in the genus that can produce azaspiracid toxins. Phycologia, 49, 169–182. 10.2216/PH09-35.1.

[ref59] Tillmann, U., Elbrächter, M., Krock, B., John, U. and Cembella, A. (2009) *Azadinium spinosum* gen. et sp. nov. (Dinophyceae) identified as a primary producer of azaspiracid toxins. Eur. J. Phycol., 44, 63–79. 10.1080/09670260802578534.

[ref60] Tillmann, U., Gottschling, M. B., Krock, B., Smith, K. F. and Guinder, V. (2019) High abundance of Amphidomataceae (Dinophyceae) during the 2015 spring bloom of the Argentinean Shelf and a new, non-toxigenic ribotype pf *Azadinium spinosum*. Harmful Algae, 84, 244–260. 10.1016/j.hal.2019.01.008. 31128809

[ref61] Tillmann, U., Gottschling, M., Guinder, V. and Krock, B. (2018a) *Amphidoma parvula* (Amphidomataceae), a new planktonic dinophyte from the Argentine Sea. Eur. J. Phycol., 53, 14–28. 10.1080/09670262.2017.1346205.

[ref62] Tillmann, U., Gottschling, M., Nézan, E. and Krock, B. (2015) First records of *Amphidoma languida* and *Azadinium dexteroporum* (Amphidomataceae, Dinophyceae) from the Irminger Sea off Iceland. Mar. Biodivers. Rec., 8, e142. 10.1017/S1755267215001128.

[ref63] Tillmann, U., Gottschling, M., Nézan, E., Krock, B. and Bilien, G. (2014) Morphological and molecular characterization of three new *Azadinium* species (Amphidomataceae, Dinophyceae) from the Irminger Sea. Protist, 165, 417–444. 10.1016/j.protis.2014.04.004. 24908198

[ref64] Tillmann, U., Kuwata, K., Cho, K., Takahashi, K., Tebben, J., Krock, B., Iwataki, M. and Kim, S. (2026) *Azadinium fusiforme*, a new species of Amphidomataceae from Korean and Japanese coastal waters lacking azaspiracid production. Eur. J. Protistol., 103, 126182. 10.1016/j.ejop.2026.126182. 41671851

[ref65] Tillmann, U., Salas, R., Gottschling, M., Krock, B., O’Driscoll, D. and Elbrächter, M. (2012a) *Amphidoma languida* sp. nov. (Dinophyceae) reveals a close relationship between *Amphidoma* and *Azadinium*. Protist, 163, 701–719. 10.1016/j.protis.2011.10.005. 22130577

[ref66] Tillmann, U., Sánchez-Ramires, S., Krock, B. and Bernales-Jiménez, A. (2017) A bloom of *Azadinium polongum* in coastal waters off Peru. Rev. Biol. Mar. Oceanogr., 52, 591–610. 10.4067/S0718-19572017000300015.

[ref67] Tillmann, U., Soehner, S., Nézan, E. and Krock, B. (2012b) First record of the genus *Azadinium* (Dinophyceae) from the Shetland Islands, including the description of *Azadinium polongum* sp. nov. Harmful Algae, 20, 142–155. 10.1016/j.hal.2012.10.001.

[ref68] Tillmann, U., Wietkamp, S., Krock, B., Tillmann, A., Voss, D. and Gu, H. (2020) Amphidomataceae (Dinophyceae) in the western Greenland area, including description of *Azadinium perforatum sp*. *nov*. Phycologia, 59, 63–88. 10.1080/00318884.2019.1670013.

[ref69] Toebe, K., Joshi, A. R., Messtorff, P., Tillmann, U., Cembella, A. and John, U. (2013) Molecular discrimination of taxa within the dinoflagellate genus *Azadinium*, the source of azaspiracid toxins. J. Plankton Res., 35, 225–230. 10.1093/plankt/fbs077.

[ref70] Tsuda, A., Sugisaki, H., Ishimaru, T., Saino, T. and Sato, T. (1993) White-noise-like distribution of the oceanic copepod *Neocalanus cristatus* in the subarctic North Pacific. Mar. Ecol. Prog. Ser., 97, 39–46. 10.3354/meps097039.

[ref71] Wang, Z., Wang, C., Wang, M., Li, W., Zhong, W., Liu, L. and Jiang, T. (2022) Diversity and community structure of eukaryotic microalgae in surface sediments in the central Bohai Sea, China, based on a metabarcoding approach. J. Oceanol. Limnol., 40, 2277–2291. 10.1007/s00343-021-0481-7.

[ref72] Welschmeyer, N. A. (1994) Fluorometric analysis of chlorophyll *a* in the presence of chlorophyll *b* and pheopigments. Limnol. Oceanogr., 39, 1985–1992. 10.4319/lo.1994.39.8.1985.

[ref73] Wietkamp, S., Krock, B., Clarke, D., Voβ, D., Salas, R., Kilcoyne, J. and Tillmann, U. (2020) Distribution and abundance of azaspiracid producing dinophyte species and their toxins in North Atlantic and North Sea waters in summer 2018. PLoS One, 15, e0235015. 10.1371/journal.pone.0235015. 32559229 PMC7304611

[ref74] Wietkamp, S., Krock, B., Gu, H., Voβ, D., Klemm, K. and Tillmann, U. (2019) Molecular detection and quantification of the azaspiracid-producing dinoflagellate *Amphidoma languida* (Amphidomataceae, Dinophyceae). J. Plankton Res., 41, 101–113. 10.1093/plankt/fby052.

[ref75] Wu, Y., Hirai, J., Zhou, F., Iwataki, M., Jiang, S., Ogawa, H., Inoue, J., Hyodo, S. et al. (2024) Diversity and biogeography of dinoflagellates in the Kuroshio region revealed by 18S rRNA metabarcoding. Front. Mar. Sci., 11, 1361452. 10.3389/fmars.2024.1361452.

