# Supplementary material for: Molecular detections of 14 amphidomatacean species (Dinophyceae) and their temperature-associated distribution in subarctic waters off eastern Hokkaido, Japan
Source: J Plankton Res. 2026 May 13;48(3):fbag029. doi: 10.1093/plankt/fbag029 (PMC13171040; doi:10.1093/plankt/fbag029)
Supplement: Supplementary_materials_fbag029 [file supplementary_materials_fbag029.pdf]

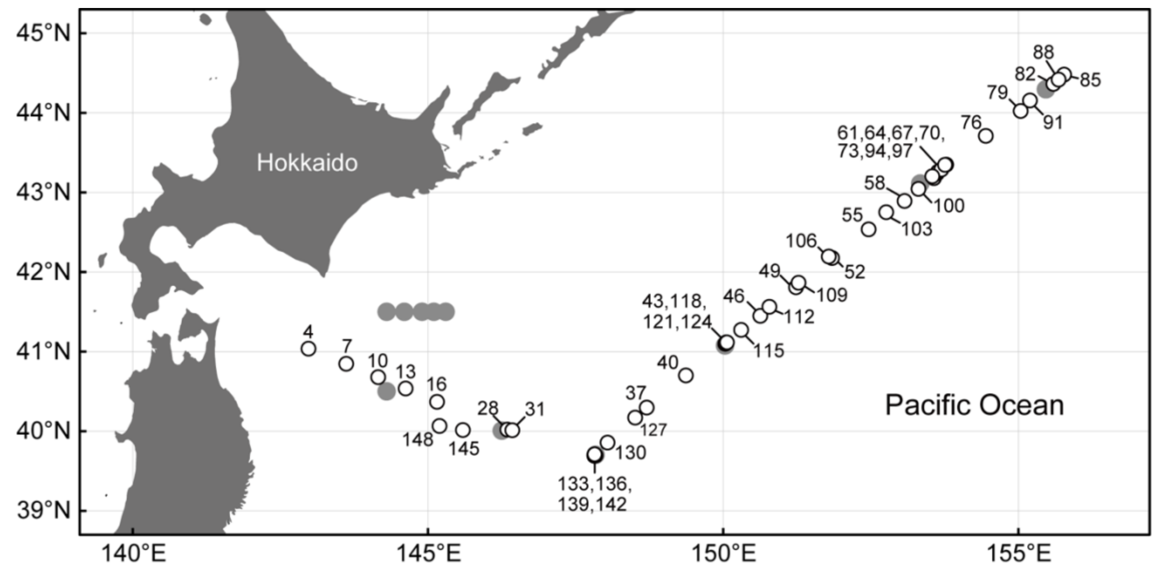

Fig. S1. Sampling locations of surface seawater by the automated seawater filtration system for environmental DNA extraction, off Hokkaido, Japan, in September 2024.

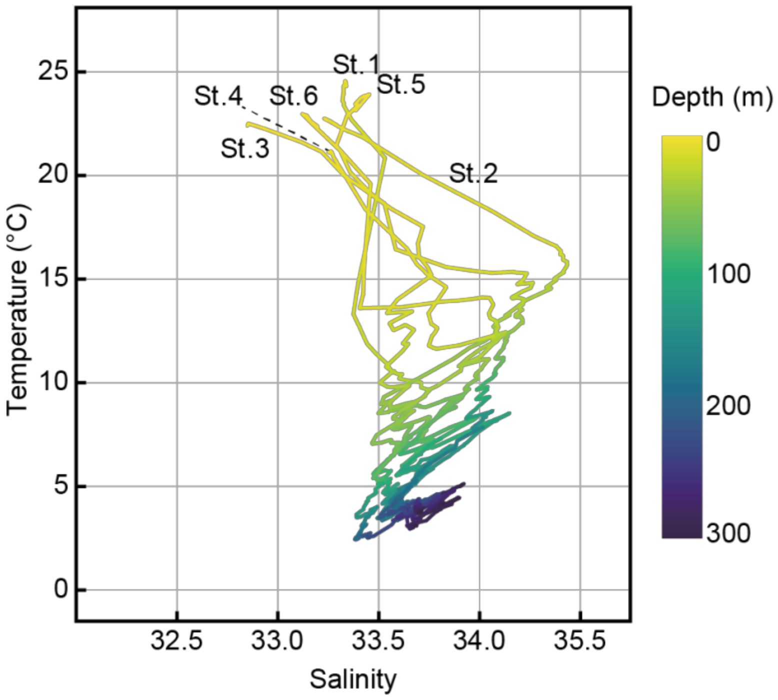

Fig. S2. Temperature-salinity diagram of 0–300 m depths collected in August 2023.

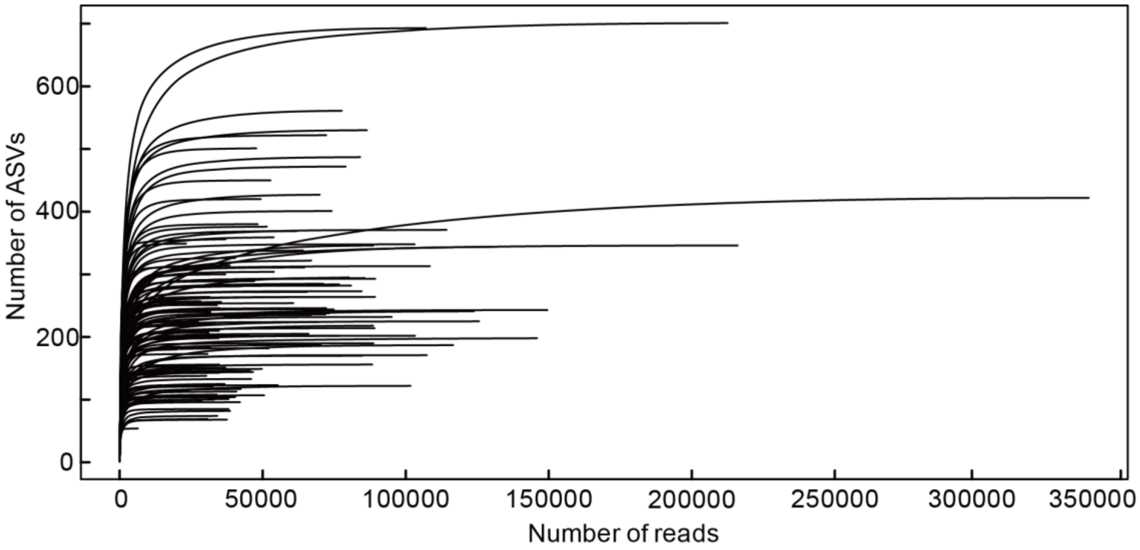

Fig. S3. The rarefaction curves of each sample based on MiSeq sequencing.

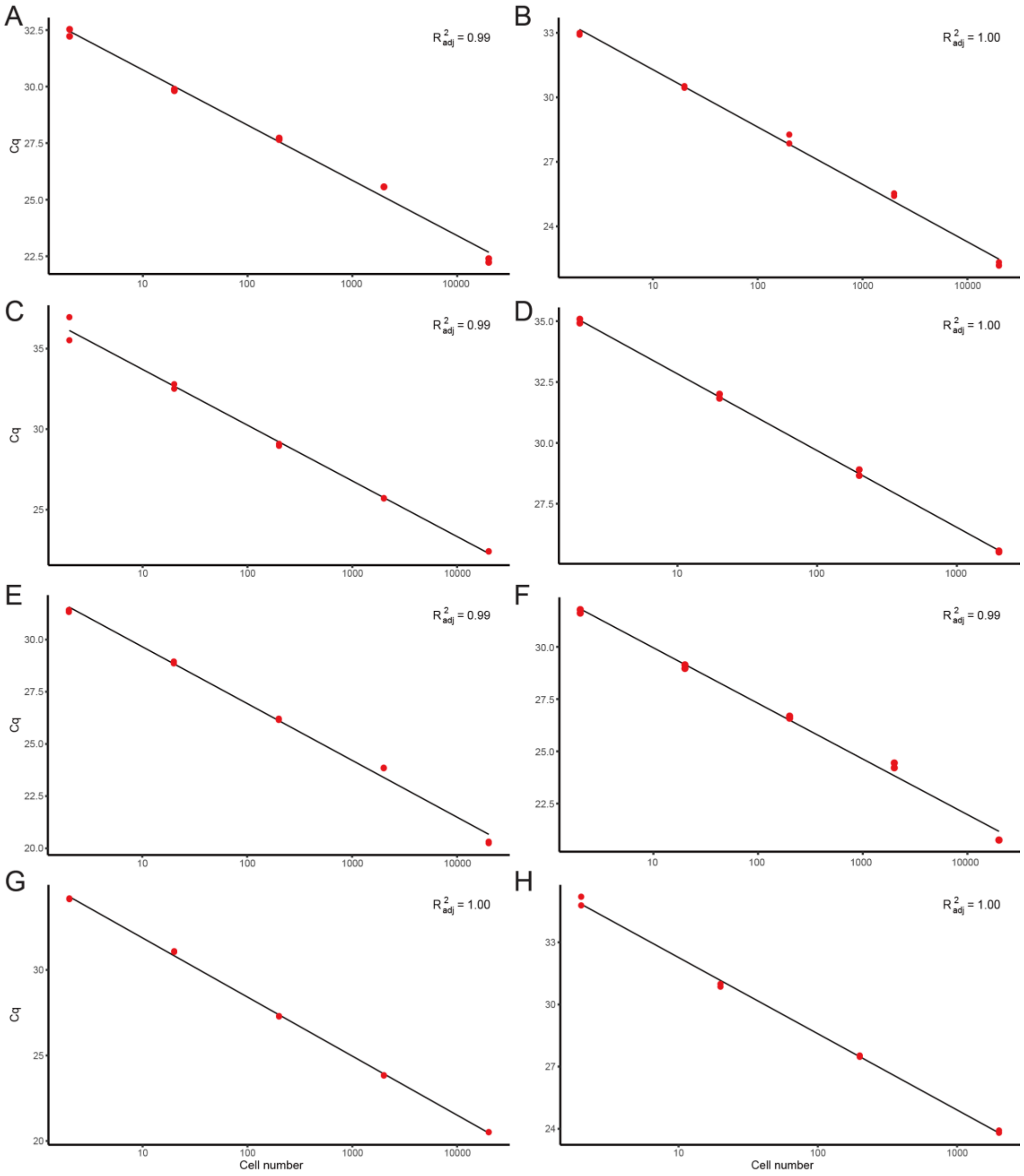

Fig. S4. Calibration curves for quantitative PCR (qPCR) assays targeting *Azadinium poporum* (A–D) and *Az. spinosum* (E–H). qPCR runs correspond to St. 1–6 (A, E), St. 7, 9, and 11 (B, F), St. 8 and 10 (C, G), and samples collected using an automated seawater filtration system (D, H). Adjusted R^2 values are shown in the upper right corner of each panel. Cq thresholds were automatically determined.

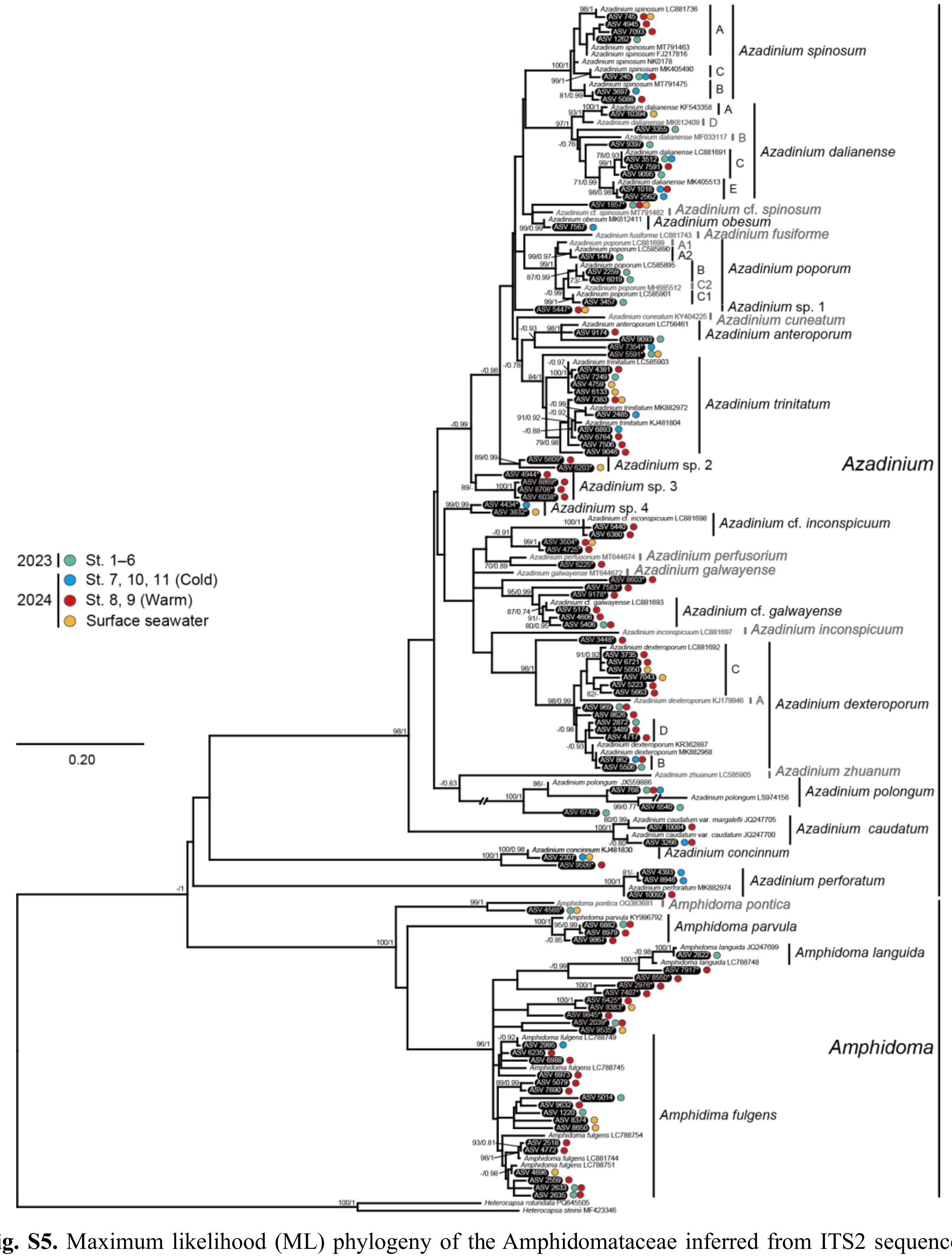

Fig. S5. Maximum likelihood (ML) phylogeny of the Amphidomataceae inferred from ITS2 sequences. Bootstrap support values (≥ 70%) of ML and posterior probabilities (≥ 0.70) of Bayesian inference are shown at nodes. ASVs detected in this study are highlighted in black. Asterisks show the unidentified ASVs not assigned to known Azadinium clades. Colored circles indicate seawater samples for each ASV.

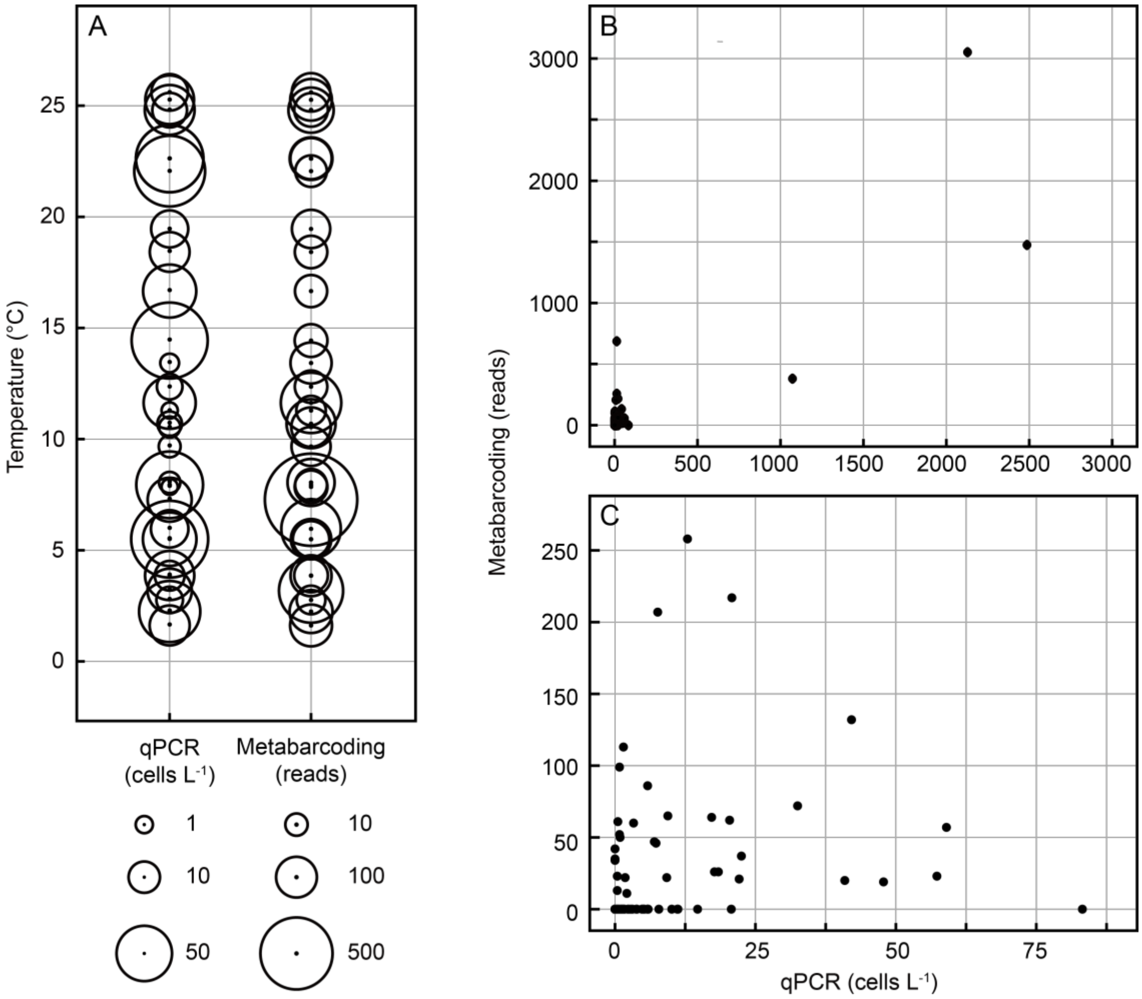

Fig. S6. Comparison between cell numbers by qPCR and ASV read numbers by eDNA metabarcoding. (A) Detected temperatures of *Azadinium spinosum* by qPCR and eDNA metabarcoding in 2024. (B) Cell numbers and ASV read numbers in both 2023 and 2024. (C) Enlarged plot of Fig. S5C.

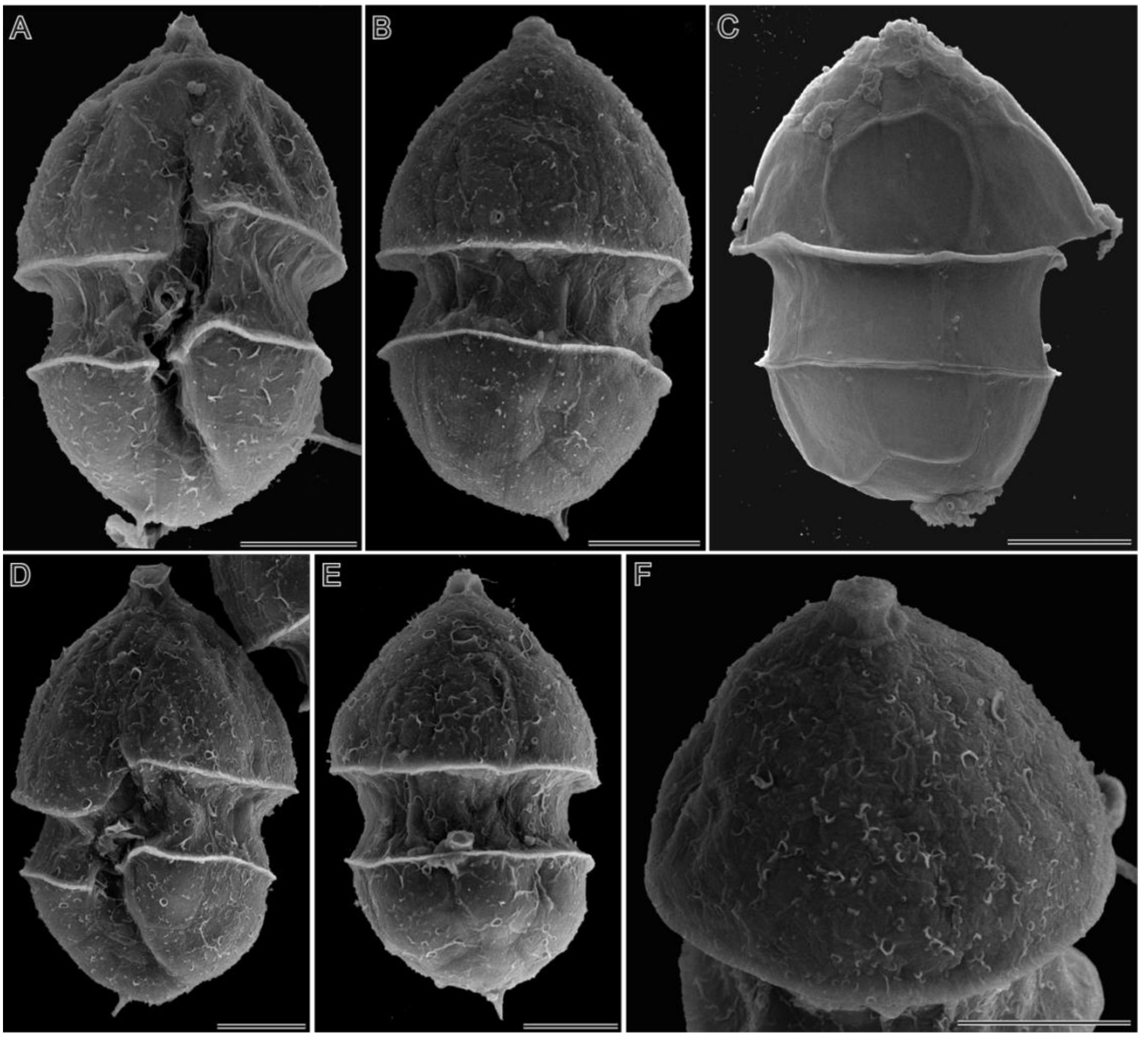

Fig. S7. Scanning electron microscopy of *Azadinium spinosum* strain NK0178. (A, D) Ventral View. (B, C, E) Dorsal view. (F) Epicone from ventral. Scale bars = 3 µm.

Table S1. Stations for vertical seawater sampling. SCM, subsurface chlorophyll maximum layer.

Station	Date (JST)	Time (JST)	Coordinates	SCM (m)	Depth of 1% surface irradiance (m)
St. 1	18 Aug. 2023	16:04	40.5°N, 144.3°E	35	28
St. 2	19 Aug. 2023	08:30	41.5°N, 144.3°E	22	35
St. 3	19 Aug. 2023	14:39	41.5°N, 144.6°E	25	33
St. 4	19 Aug. 2023	19:18	41.5°N, 144.9°E	30	30
St. 5	20 Aug. 2023	12:14	41.5°N, 145.1°E	27	45
St. 6	20 Aug. 2023	08:31	41.5°N, 145.3°E	30	36
St. 7	3 Sep. 2024	14:30	40.0°N, 146.4°E	15	43
St. 8	9 Sep. 2024	08:33	39.7°N, 147.8°E	47	90
St. 9	8 Sep. 2024	08:30	41.1°N, 150.1°E	44	61
St. 10	5 Sep. 2024	10:14	43.2°N, 153.6°E	40	53
St. 11	6 Sep. 2024	15:18	44.5°N, 155.8°E	25	43

Table S2. Stations for surface seawater sampling during the 2024 cruise.

Station	Date (JST)	Filter	Filtered volume (mL)	Coordinates	Temperature (°C)	Salinity
ASFS 1	2 Sep. 2024	Supore	768	41.2°N, 142.6°E	24.3	33.5
ASFS 4	2 Sep. 2024	Supore	5000	41.0°N, 143.0°E	23.6	33.3
ASFS 7	2 Sep. 2024	Supore	5000	40.8°N, 143.6°E	24.5	33.5
ASFS 10	2 Sep. 2024	Supore	5000	40.7°N, 144.2°E	24.6	34.4
ASFS 13	2 Sep. 2024	Supore	5000	40.5°N, 144.6°E	24.5	34.3
ASFS 16	2 Sep. 2024	Supore	5000	40.4°N, 145.2°E	25.7	34.1
ASFS 19	3 Sep. 2024	Supore	No data	40.2°N, 145.7°E	22.6	32.8
ASFS 22	3 Sep. 2024	Supore	No data	40.0°N, 146.3°E	23.2	32.9
ASFS 25	3 Sep. 2024	Omnipore	No data	40.0°N, 146.4°E	22.9	33.0
ASFS 28	3 Sep. 2024	Omnipore	4462	40.0°N, 146.4°E	23.2	32.8
ASFS 31	3 Sep. 2024	Omnipore	1572	40.0°N, 146.4°E	22.4	32.9
ASFS 37	4 Sep. 2024	Supore	5000	40.3°N, 148.7°E	24.5	33.7
ASFS 40	4 Sep. 2024	Supore	5000	40.7°N, 149.4°E	25.1	33.7
ASFS 43	4 Sep. 2024	Supore	5000	41.1°N, 150.0°E	24.9	33.8
ASFS 46	4 Sep. 2024	Supore	5000	41.5°N, 150.6°E	23.0	33.0
ASFS 49	4 Sep. 2024	Supore	5000	41.8°N, 151.2°E	23.9	33.2
ASFS 52	4 Sep. 2024	Supore	5000	42.2°N, 151.8°E	21.2	32.5
ASFS 55	4 Sep. 2024	Supore	5000	42.5°N, 152.5°E	21.7	32.7
ASFS 58	4 Sep. 2024	Supore	5000	42.9°N, 153.1°E	22.0	32.6
ASFS 61	5 Sep. 2024	Supore	4897	43.2°N, 153.6°E	22.0	32.5
ASFS 64	5 Sep. 2024	Supore	5000	43.2°N, 153.6°E	22.0	32.5
ASFS 67	5 Sep. 2024	Supore	5000	43.3°N, 153.6°E	22.0	32.5
ASFS 70	5 Sep. 2024	Supore	5000	43.3°N, 153.7°E	21.8	32.5
ASFS 73	5 Sep. 2024	Supore	5000	43.3°N, 153.8°E	21.9	32.5
ASFS 76	5 Sep. 2024	Supore	5000	43.7°N, 154.4°E	22.1	32.5
ASFS 79	6 Sep. 2024	Supore	5000	44.0°N, 155.0°E	21.3	32.4
ASFS 82	6 Sep. 2024	Supore	5000	44.4°N, 155.6°E	19.3	32.2
ASFS 85	6 Sep. 2024	Supore	5000	44.5°N, 155.8°E	19.2	32.3
ASFS 88	6 Sep. 2024	Supore	5000	44.4°N, 155.7°E	19.0	32.1
ASFS 91	6 Sep. 2024	Supore	5000	44.2°N, 155.2°E	20.2	32.4
ASFS 94	6 Sep. 2024	Supore	5000	43.3°N, 153.8°E	22.0	32.6
ASFS 97	7 Sep. 2024	Supore	5000	43.2°N, 153.5°E	22.2	32.5
ASFS 100	7 Sep. 2024	Supore	5000	43.0°N, 153.3°E	22.0	32.6
ASFS 103	7 Sep. 2024	Supore	5000	42.8°N, 152.8°E	23.2	32.9
ASFS 106	7 Sep. 2024	Supore	5000	42.2°N, 151.8°E	21.8	32.5
ASFS 109	7 Sep. 2024	Supore	5000	41.9°N, 151.3°E	23.3	33.1
ASFS 112	7 Sep. 2024	Supore	5000	41.6°N, 150.8°E	22.7	33.0
ASFS 115	7 Sep. 2024	Supore	5000	41.3°N, 150.3°E	24.6	33.6
ASFS 118	8 Sep. 2024	Supore	5000	41.1°N, 150.1°E	25.3	33.8
ASFS 121	8 Sep. 2024	Supore	5000	41.1°N, 150.1°E	25.5	33.9
ASFS 124	8 Sep. 2024	Supore	5000	41.1°N, 150.1°E	25.6	33.9
ASFS 127	8 Sep. 2024	Supore	5000	40.2°N, 148.5°E	25.6	34.0

ASFS 130	8 Sep. 2024	Supore	2786	39.9°N, 148.0°E	25.4	33.8
ASFS 133	8 Sep. 2024	Supore	3184	39.7°N, 147.8°E	25.5	33.3
ASFS 136	9 Sep. 2024	Supore	4756	39.7°N, 147.8°E	25.7	33.5
ASFS 139	9 Sep. 2024	Supore	5000	39.7°N, 147.8°E	25.8	34.4
ASFS 142	9 Sep. 2024	Supore	5000	39.7°N, 147.8°E	25.9	34.3
ASFS 145	9 Sep. 2024	Supore	5000	40.0°N, 145.6°E	23.1	34.1
ASFS 148	9 Sep. 2024	Supore	3024	40.1°N, 145.2°E	23.9	32.8

Table S3. Culture strains used for cross-reactivity test of qPCR assay. Results show either positive (+) or negative (−) for the assays.

Species	Ribo-type	Strain	Locality	*Az. poporum* assay	*Az. spinosum* assay	References
Az. dalianense	C	L3Az730	off Japan	−	−	Kuwata *et al*. 2025
Az. dexteroporum	–	LC30Az335	off Japan	−	−	Kuwata *et al*. 2025
Az. poporum	A1	LEtAz203	Japan	+	−	Kuwata *et al*. 2025
Az. poporum	A2	LAM125	Japan	+	−	Takahashi *et al*. 2021
Az. poporum	B	LEtD165	Japan	+	−	Kuwata *et al*. 2025
Az. poporum	B	LNoAz260	Japan	+	−	Kuwata *et al*. 2025
Az. poporum	B	CDAZ749	Japan	+	−	Kuwata *et al*. 2025
Az. poporum	C1	LEtAz168	Japan	+	−	Kuwata *et al*. 2025
Az. poporum	C1	CDAZ745	Japan	+	−	Kuwata *et al*. 2025
Az. spinosum	A	HrAz562	Japan	−	+	Kuwata *et al*. 2025
Az. zhuanum	–	KCAZ1	Japan	−	−	Takahashi *et al*. 2021
Am. fulgens	–	LNoAm288	Japan	−	−	Kuwata *et al*. 2024b
Am. fulgens	–	SYAz719	Japan	−	−	Kuwata *et al*. 2024b
Am. languida	–	Mex525	Mexico	−	−	Kuwata *et al*. 2024a
Heterocapsa sp.	–	KC28H105	Japan	−	−	This study

Table S4. ITS sequences and strain names of *Amphidoma* and *Azadinium* used for phylogenetic analyses.

Species	Strain	Locality	Accession number	References
Am. fulgens	GBSub03	Subic Bay, Philippines	LC788745	Kuwata *et al.* 2024b
Am. fulgens	ML489	Pulau Perhentian, Terengganu, Malaysia	LC788749	Kuwata *et al.* 2024b
Am. fulgens	NhAz554	Mutsu Bay, Japan	LC788751	Kuwata *et al.* 2024b
Am. fulgens	SYAz720	Off Miyake Is., Japan	LC788754	Kuwata *et al.* 2024b
Am. fulgens	KJG258	Sagami Bay, Japan	LC881744	Kuwata *et al.* 2025
Am. languida	SM1	Bantry Bay, Ireland	JQ247699	Tillmann *et al.* 2012
Am. languida	Mex525	Pacific Ocean, Mexico	LC788748	Kuwata *et al.* 2024a
Am. parvula	H-1E9	South Atlantic, Argentina	KY996792	Tillmann *et al.* 2018a
Am. pontica	BS6-F9	Black Sea, Turkey	OQ383681	Tillmann *et al.* 2025
Az. anteroporum	AmAz661	Mutsu Bay, Japan	LC756461	Kuwata *et al.* 2023
Az. caudatum var. *caudatum*	IFR10-332	Concarneau Bay, France	JQ247700	Nézan *et al.* 2012
Az. caudatum var. *margalefii*	AC1	Scottish coast, Scottland	JQ247705	Nézan *et al.* 2012
Az. concinnum	1C6	Irminger Sea, Greenland	KJ481830	Tillmann *et al.* 2014
Az. cuneatum	965F5	Puget Sound, USA	KY404225	Kim *et al.* 2017
Az. dalianense	AZCH02	East China Sea, China	KF543358	Luo *et al.* 2013
Az. dalianense	L3Az730	off Hamanaka, Japan	LC881691	Kuwata *et al.* 2025
Az. dalianense	IFR-ADA-01C	Concarneau Bay, France	MF033117	Luo *et al.* 2017
Az. dalianense	H-2-G7	South Atlantic, Argentina	MK405513	Tillmann *et al.* 2019
Az. dalianense	LF-09-B02	North Atlantic, Denmark	MK612409	Wietkamp *et al.* 2019
Az. dexteroporum	n.d.	Gulf of Naples, Italy	KJ179946	Percopo *et al.* 2013
Az. dexteroporum	1D12	Irminger Sea, Iceland	KR362887	Tillmann *et al.* 2015
Az. dexteroporum	LC30Az335	off Torishima Is., Japan	LC881692	Kuwata *et al.* 2025
Az. dexteroporum	AZA-2-B1	Labrador Sea, Greenland	MK882968	Tillmann *et al.* 2020
Az. fusiforme	HrAz563	Hiroshima Bay, Japan	LC881743	Tillmann *et al.* 2026
Az. galwayense	35-R7	North Atlantic, Ireland	MT644672	Salas *et al.* 2021
Az. cf. *galwayense*	KJG261	Sagami Bay, Japan	LC881693	Kuwata *et al.* 2025
Az. inconspicuum	KJG262	Sagami Bay, Japan	LC881697	Kuwata *et al.* 2025
Az. cf. *inconspicuum*	KHAz665	Mutsu Bay, Japan	LC881698	Kuwata *et al.* 2025
Az. obesum	LF-12-A09	North Atlantic, Denmark	MK612411	Wietkamp *et al.* 2019
Az. perforatum	AZA-2H	Labrador Sea, Greenland	MK882974	Tillmann *et al.* 2020
Az. perfusorium	5-B8	North Atlantic, Ireland	MT644674	Salas *et al.* 2021
Az. polongum	SHETB2	Shetland Islands, Scottland	JX559886	Tillmann *et al.* 2012
Az. polongum	N-47-01	Norwegian Sea, Norway	LS974156	Tillmann *et al.* 2018b

Az. poporum	HrAz690	Hiroshima Bay, Japan	LC881699	Kuwata *et al*. 2025
Az. poporum	MoAz592	Mutsu Bay, Japan	LC585890	Takahashi *et al*. 2021
Az. poporum	NoAz73	Mutsu Bay, Japan	LC585895	Takahashi *et al*. 2021
Az. poporum	mdd421	Sagami Bay, Japan	LC585901	Takahashi *et al*. 2021
Az. poporum	TIO427	Ionian Sea, Greece	MH685512	Luo *et al*. 2018b
Az. spinosum	3D9	North Sea, Scottland	FJ217816	Tillmann *et al*. 2009
Az. spinosum	HrAz562	Hiroshima Bay, Japan	LC881736	Kuwata *et al*. 2025
Az. spinosum	H-1-B5	South Atlantic, Argentina	MK405490	Tillmann *et al*. 2019
Az. spinosum	4-F8	North Atlantic, Ireland	MT791463	Tillmann *et al*. 2021
Az. spinosum	5-F3	North Atlantic, North Sea	MT791475	Tillmann *et al*. 2021
Az. spinosum	NK0178	Off Hokkaido, Japan	LC921252	This study
Az. cf. *spinosum*	1-H10	North Atlantic, Ireland	MT791482	Tillmann *et al*. 2021
Az. trinitatum	AsAz545	Mutsu Bay, Japan	LC585903	Takahashi *et al*. 2021
Az. trinitatum	AZA-2F	Labrador Sea, Greenland	MK882972	Tillmann *et al*. 2019
Az. trinitatum	A2D11	North Atlantic, Iceland	KJ481804	Tillmann *et al*. 2014
Az. zhuanum	KCAZ1	Tosa Bay, Japan	LC585905	Takahashi *et al*. 2021
Heterocapsa rotundata	AGSB0358	Ketch Harbour, Canada	PQ645505	Mordret *et al*. 2024
Heterocapsa steinii	UTKG1	Kiel Fjord, Germany	MF423346	Tillmann *et al*. 2017
